# Malevich’s Suprematist Composition Picture for Spin States

**DOI:** 10.3390/e21090870

**Published:** 2019-09-06

**Authors:** Vladimir I. Man’ko, Liubov A. Markovich

**Affiliations:** 1 P.N. Lebedev Physical Institute, Russian Academy of Sciences, Leninskii Prospect 53, Moscow 119991, Russia; mankovi@lebedev.ru; 2 Moscow Institute of Physics and Technology, Institutskii Per. 9, Dolgoprudny, Moscow Region 141700, Russia; 3 Russian Quantum Center, 100 Novaya St., Skolkovo, Moscow 143025, Russia; 4 V. A. Trapeznikov Institute of Control Sciences, Moscow, Profsoyuznaya 65, Moscow 117997, Russia

**Keywords:** geometry of quantum states, qudit, noncomposite quantum systems, uncertainty relations

## Abstract

This paper proposes an alternative geometric representation of single qudit states based on probability simplexes to describe the quantum properties of noncomposite systems. In contrast to the known high dimension pictures, we present the planar picture of quantum states, using the elementary geometry. The approach is based on, so called, Malevich square representation of the single qubit state. It is shown that the quantum statistics of the single qudit with some spin *j* and observables are formally equivalent to statistics of the classical system with $N^{2}-1$ random vector variables and $N^{2}-1$ classical probability distributions, obeying special constrains, found in this study. We present a universal inequality, that describes the single qudits state quantumness. The inequality provides a possibility to experimentally check up entanglement of the system in terms of the classical probabilities. The simulation study for the single qutrit and ququad systems, using the Metropolis Monte-Carlo method, is obtained. The geometrical representation of the single qudit states, presented in the paper, is useful in providing a visualization of quantum states and illustrating their difference from the classical ones.

## 1. Introduction

In modern science quantum systems are powerful resource for information processing. Many physical properties and phenomena are difficult to understand, but the geometric interpretations of quantum mechanical systems deliver an elegant way of understanding and “feeling” them. That is why the geometrical picture of physical theories draw attention in a wide range of fields from classical and quantum mechanics to the general relativity (cf. [1]). The practical implementation of large scale quantum communication networks and key distribution [2], quantum cryptography [3,4,5], quantum random number generation [6] or a quantum computer, is the main goal of quantum information science. However, for today only the simplest forms of the quantum random number generators and the quantum key distribution set-ups, exposed by statistical problems in the randomness generation, are available. That is a lot due to the fact, that for the practical use, multipartite high fidelity entangled quantum states are needed, that are challenging to control technologically. Superpositions are extraordinarily fragile, making it difficult to work with multiple qubits. Nowadays the quantum computers are based on particles that serve as qubits. However, it is possible to use qudits with more than two states simultaneously instead. The quantum computer with two 32-state qudits, would be able to perform as many operations as 10 qubits, fixing the problems arise working with 10 qubits together (see [7]).

Therefore, there are more and more papers devoted to a single *d*-level quantum systems (qudits) theory. The main interest is whether the quantum information protocols, that are today based on distributed entanglement (multy-qubit systems), can instead be realized, using the single qudit systems? What are the advantages of the these systems in comparison to the systems with subsystems? Two-particle states are determined by the matrix of the density operator $\hat{\rho }\left(1,2\right)$, which acts in the Hilbert space H. It can be represented by a tensor product $H=H_{1}⊗H_{2}$ of the Hilbert spaces of the first and second subsystems, respectively. This approach allows to construct reduced operators (density operators), describing the states of the first and second subsystems as $\hat{\rho }\left(1\right)=Tr_{2}\hat{\rho }\left(1,2\right)$ and $\hat{\rho }\left(2\right)=Tr_{1}\hat{\rho }\left(1,2\right)$. Composite systems have correlations between subsystems, so the physical meaning of entanglement for them is determined in a natural way. The presence of correlations in systems with subsystems is detected using Bell’s inequality (cf. [8,9]), which is violated for the entangled states (cf. [10]), and also entropy and information inequalities, known both for the classical distribution functions and classical observable random variables (cf. [11]), and for the density matrices of composite systems. For two- and three-partite systems, the entropy inequalities are defined as subadditivity and strong subadditivity inequalities that determine the degree of entanglement in the system (cf. [12,13]).

In [14,15,16,17] it was shown that the quantum properties of the systems without subsystems can be formulated, using the invertible mappings of indices method. The correlation properties, known for the composite systems, such as entanglement, correlation, steering and discord are formulated for the systems without subsystems in [15,18]. The quantum correlations for the system of one qudit are used to formulate a quantum contextuality in [19]. Thereby, the study of the single qudit as a resource for the quantum information is a fundamental question of quantum mechanics.

What are the possible ways to visualise large quantum states? In quantum computation and information science, the geometrical representation, based on the Bloch sphere, is commonly used. The Bloch sphere provides the representation of the quantum states of the single qubit onto a unit sphere in three real dimensions, with pure states, mapped onto the surface, and the mixed states, lying in the interior. While the Bloch sphere representation is very useful for the qubit state, it is not straightforward to generalize it easily to the qudit states. Many efforts are done to provide a more general representation that extends from the qubit to the qudit system. The Hopf fibration, providing a geometrical structure of one and two qubits, is applied in the theory of entanglement measures in [20]. In 1932 an alternative geometrical representation was proposed by Majorana in [21]. A pure state of a spin *S* is express geometrically as 2S points on the surface of a unit sphere, called the Majorana sphere. The Majorana representation is used to determine geometric phase of spins (cf. [22,23]), in studying the symmetries of spinor Bose-Einstein condensates (cf. [24,25,26]), in geometrical representation of multi-qubit entangled states (cf. [27,28]) and many other applications (cf. [29,30]). In [31,32] the pure *N*-qubit states were expresses geometrically, using the mapping that associates them with a polynomial. In [33] the study of the Majorana geometrical representation of the qutrit is presented. The geometry of separable states is studied particularly active (cf. [34,35,36,37]). The separable states are approximated by a polyhedron in $R^{N^{2}-1}$ (cf. [38]) and the probabilistic algorithm that provides a convex combination of the product states, representing them, is given in [39]. Good overview on the geometry of quantum states one can read in [1,40,41]. In our work, we want to get away from the three-dimensional representations and introduce the geometrical representation of the single qudit systems on the plane.

### 1.1. Contributions of This Paper

In this paper we would like to introduce the general geometrical picture of the qudit states, useful in understanding the nature of the entanglement phenomena in quantum systems without subsystems and as a visual characterization of the states quantumness. We use the probability representation of quantum states, introduced in [42,43]. This approach is based on quantum tomograms, that can be experimentaly measured for an arbitrary system. The single qubit is identified with the set of three probability distributions of spin projections on three perpendicular directions in space. This approach is studied and illustrated by the triangle geometry of the system, using the, so called, *Malevich square representation* in [44,45,46]. This representation, also known as *quantum suprematism approach* (after the Russian painter Kazimir Malevich (1879–1935), founder of suprematism, an art movement focused on basic geometric figures), illustrates the single qubit state in terms of three squares on the plane, obtained, using the invertible mapping of the points in Bloch sphere onto the probability distributions. The method gives a beautiful and clear geometrical interpretation for the system of one qubit. The attempts to generalize the Malevich’s geometric interpretation to the case of higher dimension quantum systems, by increasing the triples of squares with the dimension of the system are done in [46]. However, it is not clear how the tripples are related to each other, e.g., how to connect them in mosaic form reasonably? An essential difference of the presented in this paper geometric interpretation from the latter one, is an increase in the number of connected squares for the description of the higher order systems. To illustrate the quantumness in the single qudit system, we use the whole *Malevich’s suprematist composition*. Such an approach allows to obtain the new universal probability inequality in terms of the *Malevich squares* areas, connecting the density matrix elements of the quantum systems of any dimension. The upper bound of the inequality for the quantum system corresponds to a pure state just as the surface of a Bloch sphere for qubits. All the mixed states are limited by the inequality from above. At the same time, the upper limit of the inequality is different for the classical system and for the quantum one. We study the single qutrit and ququad states quantumness by finding the bounds of the this inequality. Since the amount of probabilities, characterizing the density matrix, growth sufficiently with an incising of the qudit system dimension, we use the Metropolis Monte-Carlo (MMC) method to find the bounds of the inequality. MMC is efficient when one thinks about big data problems and can be used to a quite large order qudit systems.

### 1.2. Physical Application

The uncertainty relations for both discrete and continuous variables lie in the heart of quantum theory, especially important in the context of quantum information theory. The basic concept of the uncertainty relation was introduced by Heisenberg in [47], demonstrating the impossibility of the simultaneous precise measurement of the position and momentum of an electron. Robertson [48] and Schrödinger [49] proposed an improvement of the uncertainty relation, incorporating both commutators and anticommutators of more general observables. The Robertson-Schrödinger uncertainty relation is applied for distinguishing pure and mixed states of discrete variables. Since that time in literature appeared several variations of the uncertainty relations. The generalizations and improvements are mainly focused on the uncertainty relation that are valid for the systems with more then two observables (qutrit, qudit). Namely, the Heisenberg-type uncertainty relation for three canonical observables is introduced in [50] and for arbitrary incompatible observables in [51]. The relations for more incompatible observables can be found in [52,53]. One can see, that in practice more then two incompatible observables can appear in the measurement, it is important to study uncertainty relations for many incompatible observables. The Malevich’s inequality is such uncertainty relation.

In [54,55] an experimental investigation of several Heisenberg-type uncertainty relations is reported. In our turn, we rewrite Malevich’s inequalities for the single qubit and qutrit in terms of the measurable observables to obtain inequalities similar to those, verified experimentally in the articles listed. Our Malevich’s inequality for the single qubit, compared with eight uncertainty relations known from the literature, showed one of the best lower bounds according to MMC simulation. Further, Malevich’s inequality for the qutrit system was rewritten in experimentally measurable observables, that allows its experimental verification along with several known inequalities provided in [53,54]. Thus, Malevich’s inequalities can serve as uncertainty relations for the systems of high dimensions and successfully compete with already known inequalities.

The paper is organized as follows. In Section 2 a short review on the qudit state probability description is presented. In Section 3 the quantum suprematism geometric representation of the single qubit system is given. In Section 4 we present a new polygon geometry representation of the single qudit state. The inequality on the sum of the areas of the Malevich’s squares, given by the probability distribution, associated with the triangle geometry of the single qudit state, is presented. Next, in Section 5 the MMC algorith is constructed to find the aper bound of the this inequality. The efficiency of the method is illustrated by the example of the single qutrit and ququad systems. The example of the Werner state is studied in details. In Section 7 the Malevich’s inequality is compared with several experimentally verified uncertainty relations. Conclusions and perspectives are presented in Section 8.

## 2. Parametrization of Density Matrices

In quantum physics states are represented by the density matrices on the complex Hilbert space *H* of the system. The density matrix ρ on the Hilbert space is a linear operator such that ρ≥0, Tr(ρ)=1, $\rho =\rho^{†}$. As it was mentioned in [56], the simplex of classical probabilities can be described in a quantum framework. Every probability vector is associated with a coadjoint orbit of the unitary group, acting on the dual space of its Lie algebra. The probability vector $\left(p_{1},p_{2},\ldots ,p_{N}\right)$ is rewritten as the density matrix by setting

$$\begin{array}{c} \rho \left(U,\vec{p}\right)=U\left(\begin{array}{cccc} p_{1} & 0 & \ldots & 0\\ 0 & p_{2} & \ldots & 0\\ \ldots & \ldots & \ldots & \ldots \\ 0 & 0 & \ldots & p_{N} \end{array}\right)U^{†}. \end{array}$$

The classical simplex is quantized by considering the union over it of the corresponding coadjoint orbits, each one going through the probability vector, identified with the diagonal elements of the density matrix.

**Figure 1:**
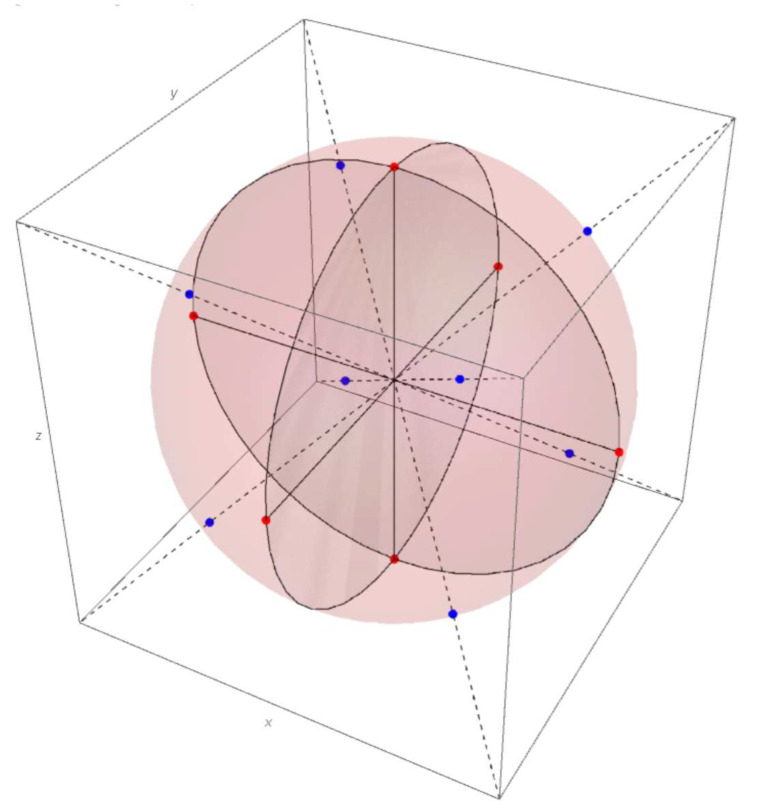
Geometric interpretation of the qubit in the probanility representation. The surface of the Bloch sphere represents the pure states of the two-dimensional quantum system, whereas the interior corresponds to the mixed states.

The main difficulty is the positivity constraint ρ≥0. This condition can not be written in polynomials for the matrices of the dimension N>4. Despite their are different matrix parametrizations, providing the positivity condition, the common view of the high order density matrices is not yet known. In [57] a good review on the recent studies on the structure and general from of the density matrix is given. In the case of N=2 the Block-sphere representation is commonly used (see Figure 1).

Three Pauli matrices

$$\begin{array}{c} \sigma_{1}=\left(\begin{array}{cc} 0 & 1\\ 1 & 0 \end{array}\right),\;\sigma_{2}=\left(\begin{array}{cc} 0 & -i\\ i & 0 \end{array}\right),\;\sigma_{3}=\left(\begin{array}{cc} 1 & 0\\ 0 & -1 \end{array}\right), \end{array}$$

together with the identity matrix $I_{2}$ form a basis of the complex vector space of Hermitian 2×2 matrices. Hence the density matrix of the single qubit state can be written as

$$\begin{array}{c} \rho_{2}=\frac{1}{2}\left(\begin{array}{cc} 1+z & x-iy\\ x+iy & 1-z \end{array}\right), \end{array}$$

in which the coefficients x,y,z are chosen such that all the eigenvalues of $\rho_{2}$ are non-negative. The positivity condition of the eigenvalues provides the set of parameters that forms a closed unit Bloch ball in $R^{3}$ with the center at 0, e.g., $x^{2}+y^{2}+z^{2}\leq 1$, holds. Hence, the parameters *x*, *y* and *z* associate the qubit states with the points either on the surface of the Block sphere, that corresponds to the pure states case, or inside the sphere for the mixed states case. That provides the geometric interpretation of the qubit states in terms of the points on the Bloch sphere.

Since we want the elements of the density matrix to have the notion of probabilities, let the density matrix be rewritten as follows

(1) $$\begin{array}{c} \rho_{2}=\left(\begin{array}{cc} p_{2} & p_{0}-\frac{1}{2}-i\left(p_{1}-\frac{1}{2}\right)\\ p_{0}-\frac{1}{2}+i\left(p_{1}-\frac{1}{2}\right) & 1-p_{2} \end{array}\right), \end{array}$$

where $p_{j}$, j=1,2,3 are the probabilities of measuring the spin −1/2 projections along the x,y,z-axes, respectively. The nonnegativity of the density matrix provides the condition

(2) $$\begin{array}{c} {\left(p_{0}-1/2\right)}^{2}+{\left(p_{1}-1/2\right)}^{2}+{\left(p_{2}-1/2\right)}^{2}\leq 1/4. \end{array}$$

This inequality impose the constraint on $p_{j}$, that means that there exist quantum correlation between the spin projections on the perpendicular directions x,y,z.

For N≥3 the following representation of density matrices is suggested

(3) $$\begin{array}{c} \rho =\frac{1}{N}I_{N}+\frac{1}{2}\sum_{j=1}^{N^{2}-1}x_{j}\lambda_{j}, \end{array}$$

where $\lambda_{j}$ are the orthogonal generators of the special unitary group SU(N) and $x_{j}\in R$ are the entries of the generalized Bloch vector. These matrices satisfy

$$\begin{array}{c} \lambda_{j}^{🟉}=\lambda_{j},\;Tr\lambda_{j}=0,\;Tr\left(\lambda_{i}\lambda_{j}\right)=2\delta_{ij},\;i,j=1,\ldots ,N^{2}-1 \end{array}$$

and the commutation and the anti-commutation relations

$$\begin{array}{c} \left[\lambda_{i}\lambda_{j}\right]=\;2i\;\;\sum_{k=1}^{N^{2}-1}f_{ijk}\lambda_{k},\left\{\lambda_{i}\lambda_{j}\right\}=\frac{4}{N}\delta_{ij}I_{N}+2\sum_{k=1}^{N^{2}-1}g_{ijk}\lambda_{k}, \end{array}$$

hold. Here $f_{ijk}$, $g_{ijk}$ are the structure constants of the Lie algebra SU(N). The generators $\lambda_{j}$, $j=1,\ldots ,N^{2}-1$ and the unit matrix $I_{n}$ form an orthogonal basis. From the properties of the generator matrices, every matrix ρ, given by (3), has the unit trace. The only thing is to find the matrices with the non-negative eigenvalues.

One of the possible explicit $\lambda_{j}$ construction can be given in terms of the generalized Gell Mann matrices. If N=3 the Gell Mann matrices are

(4) $$\begin{array}{cc} & \lambda_{1}=\left(\begin{array}{ccc} 0 & 1 & 0\\ 1 & 0 & 0\\ 0 & 0 & 0 \end{array}\right),\;\lambda_{2}=\left(\begin{array}{ccc} 0 & 0 & 1\\ 0 & 0 & 0\\ 1 & 0 & 0 \end{array}\right),\;\lambda_{3}=\left(\begin{array}{ccc} 0 & 0 & 0\\ 0 & 0 & 1\\ 0 & 1 & 0 \end{array}\right),\\ & \lambda_{4}=\left(\begin{array}{ccc} 0 & -i & 0\\ i & 0 & 0\\ 0 & 0 & 0 \end{array}\right),\;\lambda_{5}=\left(\begin{array}{ccc} 0 & 0 & -i\\ 0 & 0 & 0\\ i & 0 & 0 \end{array}\right),\;\lambda_{6}=\left(\begin{array}{ccc} 0 & 0 & 0\\ 0 & 0 & -i\\ 0 & i & 0 \end{array}\right),\\ & \lambda_{7}=\left(\begin{array}{ccc} 1 & 0 & 0\\ 0 & -1 & 0\\ 0 & 0 & 0 \end{array}\right),\;\lambda_{8}=\frac{1}{\sqrt{3}}\left(\begin{array}{ccc} 1 & 0 & 0\\ 0 & 1 & 0\\ 0 & 0 & -2 \end{array}\right). \end{array}$$

They form three sets of operators which form SU(2) algebras, namely $\left\{\lambda_{1},\lambda_{2},\lambda_{3}\right\}$, $\left\{\lambda_{4},\lambda_{5},\left(\lambda_{3}+\sqrt{3}\lambda_{8}\right)/2\right\}$ and $\left\{\lambda_{6},\lambda_{7},\left(-\lambda_{3}+\sqrt{3}\lambda_{8}\right)/2\right\}$. Using these sets of operators, one can define “artificial qubit states” (see [58]). Firstly, the matrix $\rho_{3}$ is extended to two 4×4 density matrices as

$$\begin{array}{c} \rho_{4}^{1}=\left(\begin{array}{cc} \rho_{3} & 0\\ 0 & 0 \end{array}\right),\;\rho_{4}^{2}=\left(\begin{array}{cc} 0 & 0\\ 0 & \rho_{3} \end{array}\right). \end{array}$$

The resulting matrices can be interpreted as the density matrices for the qubit systems. Using the partial trace operation, one can define four positive semidefinite matrices $\rho^{A}$, $\rho^{B}$, $\rho^{C}$ and $\rho^{D}$, that are not independent

$$\begin{array}{c} \rho^{A}=\left(\begin{array}{cc} 1-\rho_{33} & \rho_{13}\\ \rho_{31} & \rho_{33} \end{array}\right),\;\rho^{B}=\left(\begin{array}{cc} 1-\rho_{22} & \rho_{12}\\ \rho_{21} & \rho_{22} \end{array}\right),\;\rho^{C}=\left(\begin{array}{cc} 1-\rho_{11} & \rho_{13}\\ \rho_{31} & \rho_{11} \end{array}\right),\;\rho^{D}=\left(\begin{array}{cc} 1-\rho_{22} & \rho_{23}\\ \rho_{32} & \rho_{22} \end{array}\right). \end{array}$$

These qubit density matrices can be associated to four three level systems. In each system, the population of the one of the levels with the transition probability to another level determines different qubits. The off-diagonal components of these matrices are arranged in the sets given by the SU(2) algebras, namely $A:\{x_{4},x_{5}\}$, $B:\{x_{1},x_{2}\}$, $C:\{x_{4},x_{5}\}$ and $D:\{x_{6},x_{7}\}$. Hence, these four matrices can be decomposed in terms of three probabilities, given in (1). We choose the independent qubits $\rho^{A}$, $\rho^{B}$ and $\rho^{D}$ to retrieve the original 3×3 density matrix in the form

(5) $$\begin{array}{c} \rho_{3}=\left(\begin{array}{ccc} p_{3}^{A}+p_{3}^{B}-1 & B & A\\ B^{*} & 1-p_{3}^{B} & D\\ A^{*} & D^{*} & 1-p_{3}^{A} \end{array}\right), \end{array}$$

where $\left\{A,B,D\right\}=p_{1}^{A,B,D}-1/2-i\left(p_{2}^{A,B,D}-1/2\right)$. Here $p_{1,2,3}^{A,B,D}$ are the probabilities, satisfying (2). Let us rewrite the qutrit density matrix in our notations as

(6) $$\begin{array}{c} \rho_{3}=\left(\begin{array}{ccc} p_{1}+p_{2}-1 & p_{3}-1/2-i\left(p_{4}-1/2\right) & p_{5}-1/2-i\left(p_{6}-1/2\right)\\ p_{3}-1/2+i\left(p_{4}-1/2\right) & 1-p_{2} & p_{7}-1/2-i\left(p_{8}-1/2\right)\\ p_{5}-1/2+i\left(p_{6}-1/2\right) & p_{7}-1/2+i\left(p_{8}-1/2\right) & 1-p_{1} \end{array}\right), \end{array}$$

where $p_{i}\in \left[0,1\right]$, The characteristic polynomial of this matrix is $\lambda^{3}-\lambda^{2}+b\lambda +c=0$, where the coefficients b,c can be easily compute. By Viete’s formula the latter equality has three real roots if $Q^{3}-R^{2}>0$, where Q=(1−3b)/9 and R=(−2+9b+27c)/54. We are interested in nonnegative roots, that brings us to the following conditions

(7) $$\begin{array}{c} \lambda_{1}=-2\sqrt{Q}\cos\left(\phi \right)+\frac{1}{3}>0,\;\lambda_{2,3}=-2\sqrt{Q}\cos\left(\phi \pm \frac{2}{3}\pi \right)+\frac{1}{3}>0 \end{array}$$

where $\phi =\frac{1}{3}\arccos\left(R/\sqrt{Q^{3}}\right)$.

Let us introduce the qudit state, described by the 4×4 density matrix

(8) $$\begin{array}{c} \rho =\left(\begin{array}{cccc} \rho_{11} & \rho_{12} & \rho_{13} & \rho_{14}\\ \rho_{21} & \rho_{22} & \rho_{23} & \rho_{24}\\ \rho_{31} & \rho_{32} & \rho_{33} & \rho_{34}\\ \rho_{41} & \rho_{42} & \rho_{43} & \rho_{44} \end{array}\right),\;\rho^{†}=\rho ,Tr\rho =1,\rho \geq 0. \end{array}$$

This matrix can be associated with the two-qubit state with two spins j=1/2 or with the single ququad state with the spin j=3/2. In the first case we have two subsystems with the density matrices, defined by tracing with respect to subsystems degrees of freedom

(9) $$\begin{array}{cc} & \rho_{1}=\left(\begin{array}{cc} \rho_{11}+\rho_{22} & \rho_{13}+\rho_{24}\\ \rho_{31}+\rho_{42} & \rho_{33}+\rho_{44} \end{array}\right),\;\rho_{2}=\left(\begin{array}{cc} \rho_{11}+\rho_{33} & \rho_{12}+\rho_{34}\\ \rho_{21}+\rho_{43} & \rho_{22}+\rho_{44} \end{array}\right). \end{array}$$

If (8) corresponds to the single ququad state, these matrices correspond to the “artificial qubit systems”. To parametrize (8) we need 15 probabilities. Hence, using (1), (9) one gets the following elements

(10) $$\begin{array}{ccc} \rho_{11} & = & p_{3},\;\rho_{33}=p_{5},\;\rho_{22}=p_{4},\;\rho_{44}=1-p_{3}-p_{4}-p_{5},\\ \rho_{12} & = & \rho_{21}^{*}=p_{6}-1/2-i\left(p_{7}-1/2\right),\;\rho_{34}=\rho_{43}^{*}=p_{10}-1/2-i\left(p_{11}-1/2\right),\\ \rho_{13} & = & \rho_{31}^{*}=p_{1}-1/2-i\left(p_{2}-1/2\right),\;\rho_{24}=\rho_{42}^{*}=p_{8}-1/2-i\left(p_{9}-1/2\right). \end{array}$$

However, two qubit matrices can not determine the anti diagonal elements of (8). Let

$$\begin{array}{c} \rho_{14}=\rho_{41}^{*}=p_{12}-1/2-i\left(p_{13}-1/2\right),\;\rho_{23}=\rho_{32}^{*}=p_{14}-1/2-i\left(p_{15}-1/2\right), \end{array}$$

hold. The positivity conditions of this matrix are rather complicated. For detailed graphical analizess of the roots of a quartic characteristic equation, corresponding to 4×4 matrix, see [59]. For the matrices of the dimension N>4 one need to use the numerical methods to find the eigenvalues of the high order density matrices and check them on the positivity condition.

## 3. Malevich’s Squares Probability Representation of the Qubit State

In [56] the qubit density matrix was presented in terms of the three probabilities $0\leq p_{k}\leq 1$, where $p_{1}$, $p_{2}$ and $p_{3}$ are the probabilities to have in the state ρ the spin projections m=+1/2 on the directions *x*, *y* and *z*, respectively. Using this probability representation, the new triangle geometrical picture for identification of the spin −1/2 states, the Triada of Malevich’s squaresis is proposed.

### 3.1. Triangle Geometry of the Qubit State

**Figure 2:**
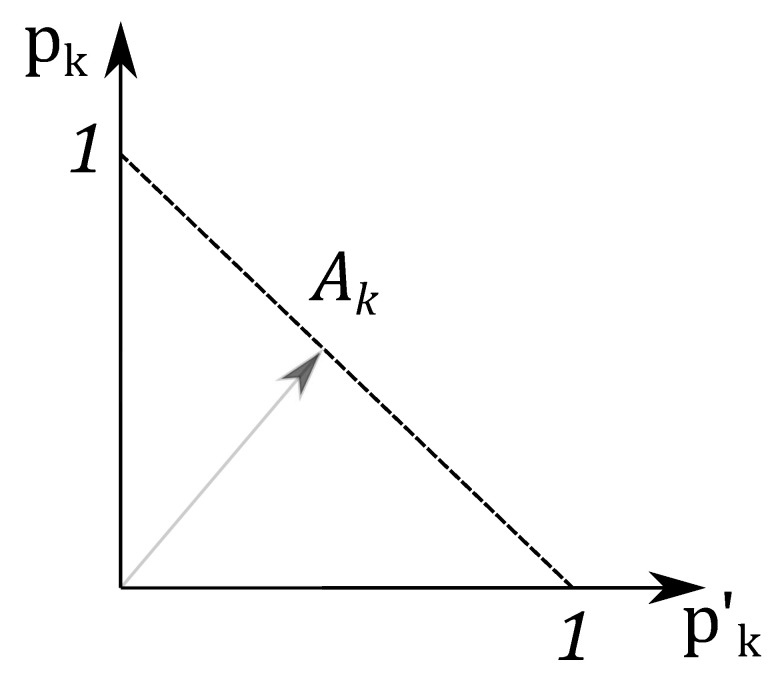
The probability vector $p_{k}$ with the end at a point $A_{k}$ on the simplex.

**Figure 3:**
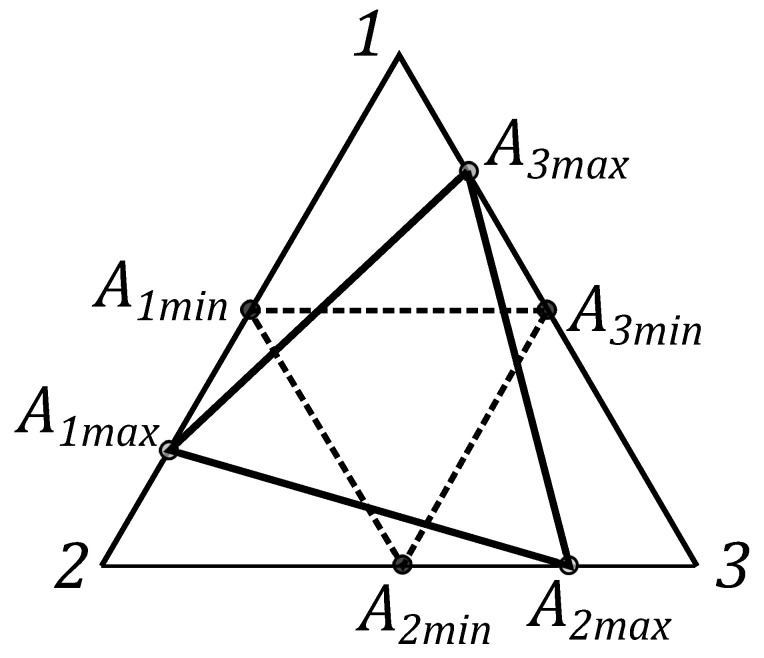
The equilateral triangle with vertices $A_{1}$, $A_{2}$ and $A_{3}$, determining the qubit state.

To illustrate the proposed triangle geometry picture for the single qubit state let us start from the statistical properties of three independent classical coins, which are associated with three probability distributions (pdf). The pdf for the first coin is given by non-negative numbers $p_{1}$ and $p_{1}^{\prime }=1-p_{1}$. The probability $p_{1}$ corresponds to the result of the experiment, when the coin look “up”. Similarly, for the second and the third coin, one has numbers $p_{2},p_{2}^{\prime }$ and $p_{3},p_{3}^{\prime }$, respectively. The pairs of probabilities can be considered as the probability vector $p_{k}={\left(p_{k},p_{k}^{\prime }\right)}^{T}$, (k=1,2,3). This vector is presented on Figure 2. Its end coincides with the point $A_{k}$ on the line, determined by the equation $p_{k}+p_{k}^{\prime }=1$, that defines the simplex with the length $\sqrt{2}$. These three simplex lines can be considered as the three sides of an equilateral triangle on the plane of equal sides $\sqrt{2}$ (see Figure 3).

One can connect points $A_{1}$, $A_{2}$ and $A_{3}$, located on simplexes, by the dashed lines and get the triangle $A_{1}A_{2}A_{2}$. We assume that $A_{k}$ are closer to the *k*th vertex of the equilateral triangle and have the distance $d_{k}=p_{k}\sqrt{2}$ from the kth vertex.

### 3.2. The Uncertainty Relation for Probabilities

The qubit state, determined by the density matrix (1), is parametrized by the three probabilities. For the latter matrix we investigate the property of the triangle $A_{1}A_{2}A_{3}$. The lengths of the triangle side $y_{k}$ is

$$\begin{array}{c} y_{k}={\left(2{\left(1-p_{k}\right)}^{2}+2p_{k+1}^{2}-2\left(1-p_{k}\right)p_{k+1}\right)}^{1/2}. \end{array}$$

**Figure 4:**
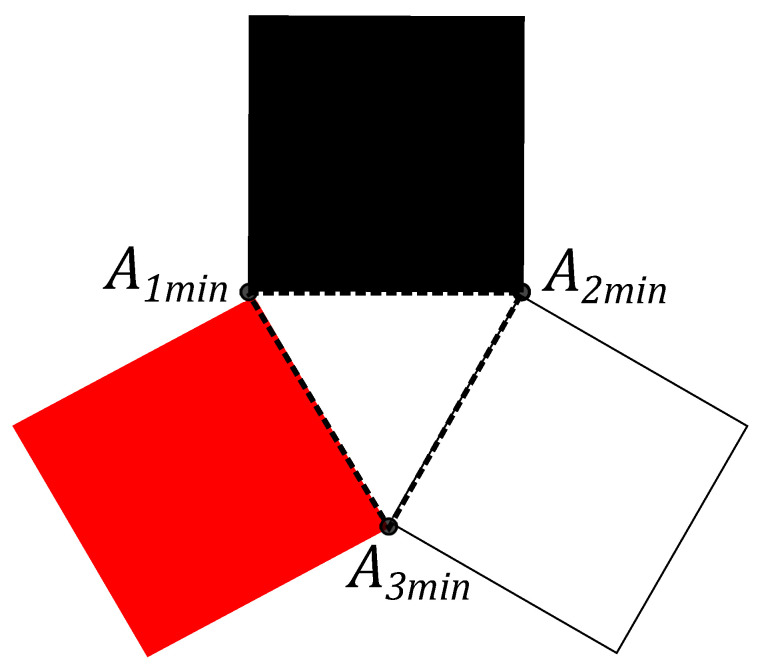
The probability vector $p_{k}=\left\{1/2,1/2,1/2\right\}$ with the end at a point $A_{kmin}$ on the simplex, S=3/2.

Three squares can constructed, analogues of the Triada of Malevich’s squares, with sides $y_{k}$, associated with the triangle $A_{1}A_{2}A_{3}$ (see Figure 4).

The sum of the areas of these three squares is expressed in terms of the three probabilities $p_{k}$ as follows

(11) $$\begin{array}{c} S\;=\;\sum_{i=1}^{3}y_{i}^{2}\;=2\;\left(\sum_{k=1}^{3}{\left(1-p_{k}\right)}^{2}+p_{k}^{2}-p_{k+1}\left(1-p_{k}\right)\right). \end{array}$$

For the classical coins, the numbers $p_{1}$, $p_{2}$, and $p_{3}$ take any values in the domain $0\leq p_{k}\leq 1$ and this sum satisfies the following inequality

(12) $$\begin{array}{c} 3/2\leq S\leq 6. \end{array}$$

**Figure 5:**
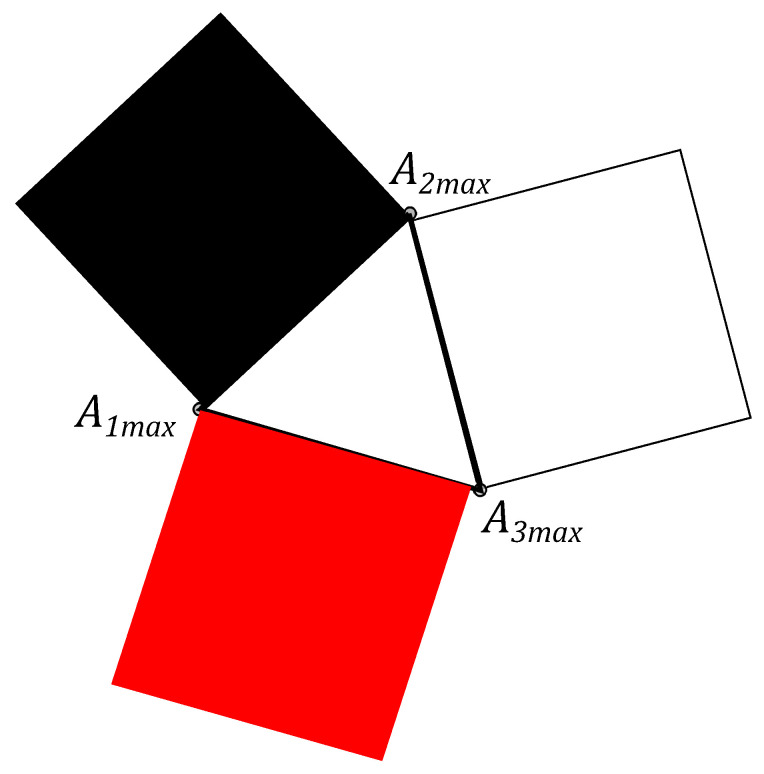
The probability vector $p_{k}=\{\left(3\pm \sqrt{3}/6,\left(3\pm \sqrt{3}\right)/6,\left(3\pm \sqrt{3}\right)/6\right\}$ with the end at a point $A_{kmax}$ on the simplex, S=3.

The points in the cube (see Figure 1) correspond to the classical coin statistics. The points on the cubes longest diagonals are extremal in the sense that the distance from the quantum states (the blue point on the Bloch sphere) to the classical states (the points in the cube’s angles vertexes) is the largest from all of the possible distances. For the quantum case the probabilities $p_{j}$ are connected by constraints, imposed by the density matrix. The detailed analysis, provided in [45,46], gives that the maximum of *S* is reached when the state is pure. The red points on the Bloch sphere (see Figure 1) correspond to $\left(p_{1},p_{2},p_{3}\right)=\{\left(0,0,1/2\right),\left(1/2,0,1/2\right),\left(1,1/2,1/2\right),\left(1/2,1,1/2\right),$ (1/2,1/2,0),(1/2,1/2,1)} and S≤2.5, holds. The blue points correspond to the case

(13) $$\begin{array}{c} p_{j}=\left(3\pm \sqrt{3}\right)/6,\;j=1,2,3,\;S\leq 3, \end{array}$$

that is the maximum value of *S* in the quantum case. Thus, the maximal side of an equilateral triangle, composed from the probabilities, that can be inscribed in a simplexes triangle, is equal to one, namely $A_{1}A_{2}=A_{2}A_{3}=A_{3}A_{1}=1$ (see Figure 5). The maximally mixed state provides the lower bound 3/2≤S, $p_{j}=1/2$, j=1,2,3. In view of this, the area of the three Malevich’s squares satisfies the inequality

$$\begin{array}{c} 3/2\leq S\leq 3, \end{array}$$

that is different from the classical one. Summing up, the properties of the area *S*, associated with the triada of the Malevich’s squares, are different for the classical system and for the quantum system states, namely, for three classical coins and for the qubit states.

## 4. Polygon Geometry of the Qudit States

Let us focus on the spin *j* system that is described by means of the Hermitian density operator $\hat{\rho }$. For the corresponding N×N matrix ρ of the latter operator, the conditions $\rho^{†}=\rho$, Trρ=1, ρ≥0, hold. In the |m〉 basis it has the following elements

$$\begin{array}{c} \rho_{mm^{\prime }}=\langle m|\hat{\rho }|m\rangle ,\;m,m^{\prime }=-j,-j+1,\ldots ,j-1,j, \end{array}$$

where N=2j+1, j=0,1/2,1,3/2,…. That means that the density matrix can be parametrized by $N^{2}-1$ parameters. According to the previous section we think about $N^{2}-1$ independent classical coins, which are associated with $N^{2}-1$ pdfs. The $N^{2}-1$ probability vectors $p_{k}={\left(p_{k},p_{k}^{\prime }\right)}^{T}$, $\left(k=1,\ldots ,N^{2}-1\right)$ can be considered. Following the line of the qubit example, one needs to connect $N^{2}-1$ simplexes to from an $N^{2}-1$-angle polygon with the side $\sqrt{2}$ and apexes marked by $k=1,\ldots ,N^{2}-1$. If to connect every apex of the polygon with the zero point of the coordinate axes, the $N^{2}-1$-sectors with angle $\beta =∠\left(1,0,2\right)=2\pi /\left(N^{2}-1\right)$ and the angle γ=∠(1,2,3)=π−β, hold.

The polygon formed by the points $A_{k}$ has the sides defined as follows

(14) $$\begin{array}{c} \;\;\;\;\;\;\;\;A_{k}A_{k+1}\;=\;\;\sqrt{2{\left(1-p_{k}\right)}^{2}+2p_{k+1}^{2}\;\;-4\left(1-p_{k}\right)p_{k+1}\;\cos\gamma }. \end{array}$$

**Figure 6:**
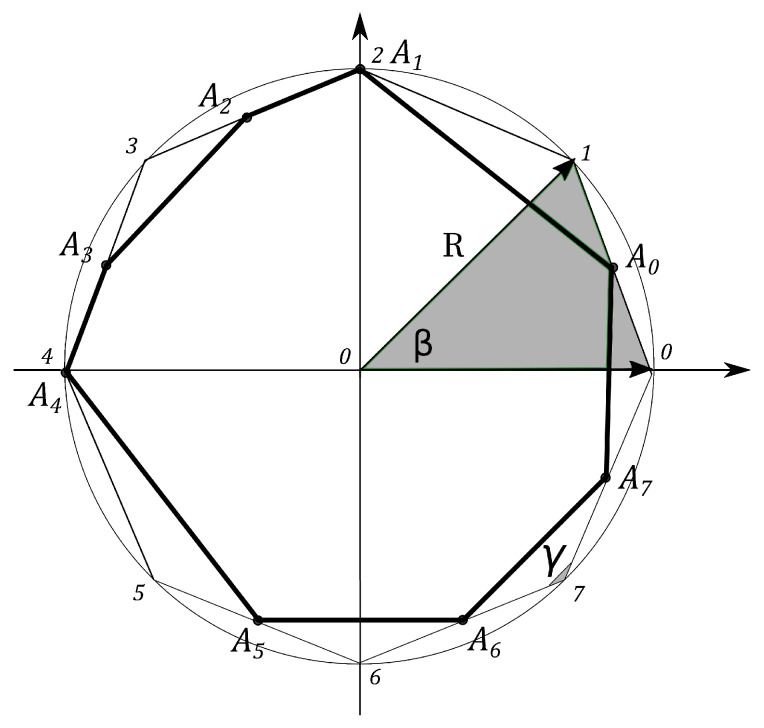
The eight angle polygon with vertices $A_{k}$, k=1,…,8, determining the qutrit state.

**Figure 7:**
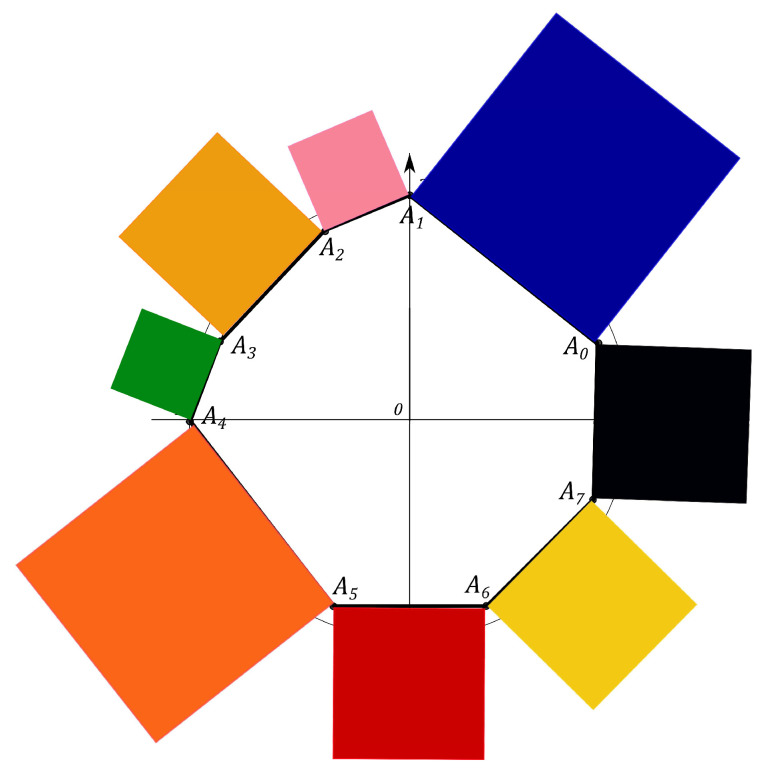
The Suprematist Composition, determining the qutrit state.

$N^{2}-1$ squares are constructed, counterparts of the *Malevich’s Suprematist Composition*, with sides $A_{k}A_{k+1}$, associated with the polygon $A_{1}A_{2}\ldots A_{N^{2}-1}$. For example, the eight angle polygon and the Malevich’s Suprematist Composition for the single qutrit are shown in Figure 6 and Figure 7. The sum of there areas is the following

(15) $$\begin{array}{c} S=\;2\left(\sum_{k=1}^{N^{2}-1}{\left(1-p_{k}\right)}^{2}+p_{k}^{2}-2p_{k+1}\left(1-p_{k}\right)\cos\gamma \right), \end{array}$$

where $p_{N^{2}}=p_{1}$.

However, in the quantum case, an additional condition on the eigenvalues of the density matrix is imposed. Since it must be non-negative, the probabilities that parametrize the density matrix, are connected by additional constraints and the value of the maximum area changes, e.g.,

$$\begin{array}{c} S<S_{qmax}\leq S_{max}. \end{array}$$

Further, we find these maxima for the single qutrit and ququad systems.

### Parametrization of Unitary Matrices

To find the maximum value of (15) one has to check all the possible probability combinations, satisfying the density matrix positivity condition. Finding the eigenvalues of the matrix and checking there positivity is technically hard and is not scalable well. To solve this problem, we use the fact that the unitary transformation does not change the physics of the system, namely it transforms one description of the system to another physically equivalent description. Hence, if we use the transformation $\rho \left[1\right]=U\rho \left[0\right]U^{†}$, where *U* is the unitary matrix, for the density matrix ρ(0), we get another density matrix with the same spectra. Changing the parameters of the unitary rotation matrix and the starting density matrix ρ(0) we can estimate the maximum of *S*. Using the initial density matrix, formed by the starting set of the probabilities and using the unitary rotation, it is possible to find all the possible density matrices of the desired dimension and extract the new probability vectors to find the maximum of *S*. Further we will discuss the algorithm in details.

The aim of this section is to present a parametization discribed in [60,61] of the unitary matrices that is used in the algorithm of the maximum searching, presented in the next section. The advantage of this parametrization method is that it is recursive. This fact allows to parametrize the N×N matrix through the parametrization of the lower dimension unitary matrices.

The unitary matrix of the dimension 2×2 has the following form

(16) $$\begin{array}{c} U_{2}=\left(\begin{array}{cc} he^{i\phi_{22}} & \sqrt{1-h^{2}}e^{i\phi_{23}}\\ \sqrt{1-h^{2}}e^{i\phi_{32}} & -he^{i(\phi_{23}+\phi_{32}-\phi_{22})} \end{array}\right), \end{array}$$

where h∈(0,1), $\phi_{23},\phi_{32},\phi_{22}\in \left[0,2\pi \right)$. Let $\rho_{2}\left[k\right]$ be in the form (1), depending from the probability vector $p\left[k\right]=(p_{0}\left[k\right],p_{1}\left[k\right],p_{2}\left[k\right])$, where *k* is the probability configuration number. After the unitary transformation, given by $U_{2}$, the density matrix has the following form

(17) $$\begin{array}{c} U_{2}\rho_{2}\left[k\right]U_{2}^{-1}\;=\;\left(\begin{array}{cc} v_{00}\left(p\left[k\right]\right) & v_{01}\left(p\left[k\right]\right)\\ v_{10}^{*}\left(p\left[k\right]\right) & v_{11}\left(p\left[k\right]\right) \end{array}\right)\equiv \rho_{2}\left[k+1\right]. \end{array}$$

Hence the transformed vector is

$$\begin{array}{c} p_{0}\left[k+1\right]=2Re\left[v_{01}\left(p\left[k\right]\right)\right],\;p_{1}\left[k+1\right]=-2Im\left[v_{01}\left(p\left[k\right]\right)\right],\;p_{2}\left[k+1\right]=2v_{00}\left(p\left[k\right]\right)-1. \end{array}$$

In case of a 3×3 matrix, we use the unitary rotation matrix in the form

(18) $$\begin{array}{c} U_{3}=\left(\begin{array}{ccc} be^{-i\phi_{12}} & \sqrt{1-b^{2}} & 0\\ c\sqrt{1-b^{2}}e^{-i\phi_{13}} & -bce^{-i(\phi_{12}-\phi_{13})} & -\sqrt{1-c^{2}}e^{-i\phi_{13}}\\ \sqrt{\left(1-b^{2}\right)\left(1-b^{2}\right)}e^{-i\phi_{31}} & -b\sqrt{1-c^{2}}e^{i(\phi_{12}-\phi_{14})} & ce^{-i\phi_{14}} \end{array}\right), \end{array}$$

where b,c∈(0,1), $\phi_{12},\phi_{13},\phi_{13}\in \left[0,2\pi \right)$. Let $\rho_{3}\left[k\right]$ be in the form (6), depending from the probability vector $p\left[k\right]=(p_{0}\left[k\right],p_{1}\left[k\right],\ldots ,p_{7}\left[k\right])$, where *k* is the number of the probabilities configuration, corresponding to the unitary rotation step, k=0,1,2,3,…. After the unitary transformation (18), the matrix $\rho_{3}\left[k+1\right]$ has the following form

(19) $$\begin{array}{c} U_{3}\rho_{3}\left[k\right]U_{3}^{-1}=\left(\begin{array}{ccc} r_{00}\left(p\left[k\right]\right) & r_{01}\left(p\left[k\right]\right) & r_{02}\left(p\left[k\right]\right)\\ r_{10}^{*}\left(p\left[k\right]\right) & r_{11}\left(p\left[k\right]\right) & r_{12}\left(p\left[k\right]\right)\\ r_{20}^{*}\left(p\left[k\right]\right) & r_{21}^{*}\left(p\left[k\right]\right) & r_{22}\left(p\left[k\right]\right) \end{array}\right). \end{array}$$

The transformed vector is

$$\begin{array}{cc} & p_{0}\left[k+1\right]=1-2r_{22}\left(p\left[k\right]\right),\;p_{1}\left[k+1\right]=1-r_{11}\left(p\left[k\right]\right),\;p_{2}\left[k+1\right]=Re\left[r_{01}\left(p\left[k\right]\right)\right]+1/2,\\ & p_{3}\left[k+1\right]=-Im\left[r_{01}\left(p\left[k\right]\right)\right]+1/2,\;p_{4}\left[k+1\right]=Re\left[r_{02}\left(p\left[k\right]\right)\right]+1/2,\\ & p_{5}\left[k+1\right]=-Im\left[r_{02}\left(p\left[k\right]\right)\right]+1/2,p_{6}\left[k+1\right]=Re[r_{12}\left(p\left[k\right]\right)+1/2,p_{7}\left[k+1\right]=-Im\left[r_{12}\left(p\left[k\right]\right)\right]+1/2. \end{array}$$

To parametrize N×N we use the procedure introduced in [61]. The matrix *S* is partitioned in blocks

$$\begin{array}{c} U_{4}=\left(\begin{array}{cc} A & B\\ C & D \end{array}\right). \end{array}$$

The element *A* is chosen as $A=ae^{i\phi_{11}}$, a∈(0,1), $\phi_{11}\in \left[0,2\pi \right)$. Then the latter matrix can be rewritten in the following form

(20) $$\begin{array}{c} U_{4}=\left(\begin{array}{cc} ae^{i\phi_{11}} & \sqrt{1-a^{2}}U\\ \sqrt{1-a^{2}}V & -ae^{-i\phi_{11}}UV+XMY^{🟉} \end{array}\right), \end{array}$$

where $U,V\in C^{N-1}$ are row and column vectors, respectively, lying on the complex unit sphere, i.e.,

$$\begin{array}{c} \sum_{i=1}^{N-1}|u_{i}{|}^{2}=\sum_{i=1}^{N-1}{\left|v_{i}\right|}^{2}=1 \end{array}$$

and *X*, *Y* are the unitary matrices such that

$$\begin{array}{c} X^{🟉}D_{V^{🟉}}X=P,\;Y^{🟉}D_{U}Y=P, \end{array}$$

where $D_{V^{🟉}}=\sqrt{I_{N-1}-VV^{🟉}}$, $D_{U}=\sqrt{I_{N-1}-U^{🟉}U}$ and

$$\begin{array}{c} P=\left(\begin{array}{cc} 0 & 0\\ 0 & I_{N-2} \end{array}\right),\;M=\left(\begin{array}{cc} 0 & 0\\ 0 & S_{N-2} \end{array}\right), \end{array}$$

where $S_{N-2}$ is the unitary N−2×N−2 matrix.

This procedure was applied in case of the unitary U(4) group in [61], but the final matrix is introduced in [60] with some misprints. The corrected matrix elements of

$$\begin{array}{c} U_{4}=\left(\begin{array}{cccc} u_{11} & u_{12} & u_{13} & u_{14}\\ u_{21} & u_{22} & u_{23} & u_{24}\\ u_{31} & u_{32} & u_{33} & u_{34}\\ u_{41} & u_{42} & u_{43} & u_{44} \end{array}\right), \end{array}$$

are the following

$$\begin{array}{lll} u_{11} & = & ae^{i\phi_{11}},\;u_{21}=d\sqrt{1-a^{2}}e^{i\phi_{21}},\;u_{12}=\sqrt{1-a^{2}}be^{i\phi_{12}},\;u_{31}=\sqrt{\left(1-a^{2}\right)\left(1-d^{2}\right)}fe^{i\phi_{31}},\\ u_{41} & = & \sqrt{\left(1-a^{2}\right)\left(1-d^{2}\right)\left(1-f^{2}\right)}e^{i\phi_{41}},\;u_{13}=\sqrt{\left(1-a^{2}\right)\left(1-b^{2}\right)}ce^{i\phi_{13}},\\ u_{14} & = & \sqrt{\left(1-a^{2}\right)\left(1-b^{2}\right)\left(1-c^{2}\right)}e^{i\phi_{14}},\;u_{22}=-abde^{i(\phi_{11}+\phi_{12}+\phi_{21})}+\sqrt{\left(1-b^{2}\right)\left(1-d^{2}\right)}e^{iy}x,\\ u_{23} & = & -acd\sqrt{1-b^{2}}e^{i(-\phi_{11}+\phi_{13}+\phi_{21})}-bc\sqrt{1-d^{2}}e^{-i(\phi_{12}-\phi_{13})+iy}x-\sqrt{\left(1-c^{2}\right)\left(1-d^{2}\right)\left(1-x^{2}\right)}e^{i\phi_{13}+iz},\\ u_{24} & = & -ad\sqrt{\left(1-b^{2}\right)\left(1-c^{2}\right)}e^{i(-\phi_{11}+\phi_{14}+\phi_{21})}-bx\sqrt{\left(1-c^{2}\right)\left(1-d^{2}\right)}e^{-i(\phi_{12}-\phi_{14}+y)}\\ & + & c\sqrt{1-d^{2}}e^{i(\phi_{14}+z)}\sqrt{1-x^{2}},\\ u_{32} & = & -abf\sqrt{1-d^{2}}e^{i(-\phi_{11}+\phi_{12}+\phi_{31})}+\sqrt{1-b^{2}}\left(-dfxe^{i(-\phi_{21}+\phi_{31}+y)}-e^{i(\phi_{31}+w)}\sqrt{\left(1-f^{2}\right)\left(1-x^{2}\right)}\right),\\ u_{33} & = & -afc\sqrt{\left(1-b^{2}\right)\left(1-d^{2}\right)}e^{i(-\phi_{11}+\phi_{13}+\phi_{31})}-\sqrt{1-c^{2}}e^{i\phi_{13}}(e^{i(\phi_{31}+w-y+z)}x\sqrt{1-f^{2}}\\ & - & dfe^{i(-\phi_{21}+\phi_{31}+z)}\sqrt{1-x^{2}})-bce^{-i(\phi_{12}-\phi_{13})}\left(-dfxe^{i(-\phi_{21}+\phi_{31}+y)}-e^{i(\phi_{31}+w)}\sqrt{\left(1-f^{2}\right)\left(1-x^{2}\right)}\right),\\ u_{34} & = & -af\sqrt{\left(1-b^{2}\right)\left(1-c^{2}\right)\left(1-d^{2}\right)}e^{i(-\phi_{11}+\phi_{14}+\phi_{31})}+ce^{i\phi_{14}}(e^{i(\phi_{31}+w-y+z)}x\sqrt{1-f^{2}}\\ & - & dfe^{i(-\phi_{21}+\phi_{31})+iz}\sqrt{1-x^{2}})-b\sqrt{1-c^{2}}e^{-i(\phi_{12}-\phi_{14})}\left(-dfxe^{i(-\phi_{21}+\phi_{31}+y)}-e^{i(\phi_{31}+w)}\sqrt{\left(1-f^{2}\right)\left(1-x^{2}\right)}\right),\\ u_{42} & = & -abe^{i(-\phi_{11}+\phi_{12}+\phi_{41})}\sqrt{\left(1-d^{2}\right)\left(1-f^{2}\right)}+\sqrt{1-b^{2}}\left(-dx\sqrt{1-f^{2}}e^{i(-\phi_{21}+\phi_{41}+y)}+f\sqrt{1-x^{2}}e^{i(\phi_{41}+w)}\right),\\ u_{43} & = & -ac\sqrt{1-b^{2}}e^{i(\phi_{11}+\phi_{13}+\phi_{41})}\sqrt{\left(1-d^{2}\right)\left(1-f^{2}\right)}-bce^{-i(\phi_{12}-\phi_{13})}(-dx\sqrt{1-f^{2}}e^{i(-\phi_{21}+\phi_{41}+y)}\\ & + & f\sqrt{1-x^{2}}e^{i(\phi_{41}+w)})+\sqrt{1-c^{2}}e^{i\phi_{13}}\left(fxe^{i(\phi_{41}+w-y+z)}+de^{i(-\phi_{21}+\phi_{41}+z)}\sqrt{\left(1-f^{2}\right)\left(1-x^{2}\right)}\right),\\ u_{44} & = & -a\sqrt{\left(1-b^{2}\right)\left(1-c^{2}\right)}e^{i(-\phi_{11}+\phi_{14}+\phi_{41})}\sqrt{\left(1-d^{2}\right)\left(1-f^{2}\right)}-b\sqrt{1-c^{2}}e^{-i(\phi_{12}-\phi_{14})}\\ & \cdot & (-dx\sqrt{1-f^{2}}e^{i(-\phi_{21}+\phi_{41}+y)}+e^{i\phi_{41}+iw}f\sqrt{1-x^{2}}+ce^{i\phi_{14}}(-fxe^{i(\phi_{41}+w-y+z)}\\ & - & de^{i(-\phi_{21}+\phi_{41}+z)}\sqrt{\left(1-f^{2}\right)\left(1-x^{2}\right)}), \end{array}$$

where

$$\begin{array}{c} \alpha ={\left(f^{2}+d^{2}-f^{2}d^{2}\right)}^{-1/2},\;\beta ={\left(b^{2}+c^{2}-b^{2}c^{2}\right)}^{-1/2},\\ a,b,c,d,f,g,x\in (0,1),\;\phi ,y,w,z\in [0,2\pi ). \end{array}$$

Let $\rho_{4}\left[k\right]$ be in the form (11), depending from the probability vector $p\left[k\right]=(p_{0}\left[k\right],p_{1}\left[k\right],\ldots ,p_{14}\left[k\right])$, where *k* is the probability configuration number. After the unitary transformation, the density matrix has the following form

$$\begin{array}{c} U_{4}\rho_{4}\left[k\right]U_{4}^{-1}\;\;=\;\;\left(\begin{array}{cccc} w_{00}\left(p\left[k\right]\right) & w_{01}\left(p\left[k\right]\right) & w_{02}\left(p\left[k\right]\right) & w_{03}\left(p\left[k\right]\right)\\ w_{10}^{*}\left(p\left[k\right]\right) & w_{11}\left(p\left[k\right]\right) & w_{12}\left(p\left[k\right]\right) & w_{13}\left(p\left[k\right]\right)\\ w_{20}^{*}\left(p\left[k\right]\right) & w_{21}^{*}\left(p\left[k\right]\right) & w_{22}\left(p\left[k\right]\right) & w_{23}\left(p\left[k\right]\right)\\ w_{30}^{*}\left(p\left[k\right]\right) & w_{31}^{*}\left(p\left[k\right]\right) & w_{32}\left(p\left[k\right]\right) & w_{33}\left(p\left[k\right]\right) \end{array}\right). \end{array}$$

Hence the transformed vector is

$$\begin{array}{ll} & p_{0}\left[k+1\right]=-Im\left(w_{03}\left(p\left[k\right]\right)\right)+1/2,\;p_{1}\left[k+1\right]=Re\left(w_{02}\left(p\left[k\right]\right)\right)+1/2,\\ & p_{2}\left[k+1\right]=-Im\left(w_{02}\left(p\left[k\right]\right)\right)+1/2,\;p_{14}\left[k+1\right]=Re\left(w_{03}\left(p\left[k\right]\right)\right)+1/2\\ & p_{4}\left[k+1\right]=w_{11}\left(p\left[k\right]\right),\;p_{5}\left[k+1\right]=w_{22}\left(p\left[k\right]\right),\;p_{3}\left[k+1\right]=w_{00}\left(p\left[k\right]\right),\\ & p_{6}\left[k+1\right]=Re\left(w_{01}\left(p\left[k\right]\right)\right)+1/2,\;p_{7}\left[k+1\right]=-Im\left(w_{01}\left(p\left[k\right]\right)\right)+1/2,\\ & p_{8}\left[k+1\right]=Re\left(w_{13}\left(p\left[k\right]\right)\right)+1/2,\;p_{9}\left[k+1\right]=-Im\left(w_{13}\left(p\left[k\right]\right)\right)+1/2,\\ & p_{10}\left[k+1\right]=Re\left(w_{23}\left(p\left[k\right]\right)\right)+1/2,\;p_{11}\left[k+1\right]=-Im\left(w_{23}\left(p\left[k\right]\right)\right)+1/2,\\ & p_{12}\left[k+1\right]=Re\left(w_{12}\left(p\left[k\right]\right)\right)+1/2,\;p_{13}\left[k+1\right]=-Im\left(w_{12}\left(p\left[k\right]\right)\right)+1/2. \end{array}$$

In the next section we will use this vector to find the maximum of (15) for the different qudit states.

## 5. Materials and Methods

### Metropolis Monte Carlo Maximum Search

Monte-Carlo (MC) method is a general name for a variety of stochastic techniques. It is based on the use of the random numbers and the probability statistics to investigate problems in many areas like economics, nuclear physics or flow of traffic. In this paper we use the Metropolis MC (MMC) that is generally used in statistical physics to solve the Ising problem, where one searches the spin configuration that provides the minimum energy of the system. The MMC was developed in present form by Metropolis, Ulam and Neumann during their work on Manhattan project (study of neutron diffusion) (cf. [62]).

The approach that is used in MMC algorithm uses random walk in the phase space with transition probability to go from the state *m* to the state *n*. It is equal to 1 if the move decrease the energy ($\Delta E_{nm}<0$). If the move increase the energy ($\Delta E_{nm}>0$) then it is accepted with a probability, defined by the ratio of the probabilities of initial and final states P(n)/P(m).

We start from setting up a random walk through the configurational space. The “time” *t* is the number of iterations of the procedure (not real time). P(m,t) is the probability of being in configuration *m* at time *t*, P(n,t) the probability of being in configuration *n* at time *t*, W(m→n,t) is the probability of going from the state *m* to the state *n* per unit time (transition probability). Then we have a sufficient (but not necessary) detailed balance condition W(n→m)P(n,t)=W(m→n)P(m,t).

In this paper we want to find the probability vector that maximizes *S*, given by (15). To this end the following steps must be done.

- Initialize the starting density matrix in the diagonal form
  $$\begin{array}{ccc} \rho_{N}^{diag} & = & \left(\begin{array}{cccccc} {\tilde{p}}_{1} & 0 & 0 & 0 & \ldots & 0\\ 0 & {\tilde{p}}_{2} & 0 & 0 & \ldots & 0\\ 0 & 0 & {\tilde{p}}_{3} & 0 & \ldots & 0\\ \vdots & \vdots & \vdots & \ddots & \vdots & \vdots \\ 0 & 0 & 0 & 0 & 0 & 1-\sum_{i=1}^{N}{\tilde{p}}_{i} \end{array}\right),\;0\leq {\tilde{p}}_{i}\leq 1. \end{array}$$
  and the unitary rotation matrix from the SU(N) group, for example, parametrized according to the recursive method, described above.
- Perform this relaxation step until freezing of the maximum *S* occurs:
  1. Randomly select one of the unitary matrix parameters or one of the diagonal parameters ${\tilde{p}}_{i}$ of $\rho_{N}^{diag}$. Slightly change it using the random generator. It is necessary to check that the changed matrix parameters are not beyond their limits, and the elements of the diagonal density matrix satisfy $0\leq {\tilde{p}}_{i}\leq 1$ and does not change the sign of the density matrix.
  2. Using the changed unitary matrix or the diagonal density matrix, perform the rotation, i.e., $\rho \left[1\right]=U\rho_{N}^{diag}\left[0\right]U^{†}$.
  3. Using the general view of the density matrix, express the new trial probabilities through the elements of the rotated matrix ρ[1]. Check that trial probabilities are $0\leq p_{tr}\leq 1$, hold.
  4. Perform the Metropolis step:
    - Generate a uniformly distributed random number ξ.
    - If $S\left(p_{tr}\right)>S\left(p\right)$ or $\xi \leq {\left(S\left(p_{tr}\right)/S\left(p\right)\right)}^{\beta }$
      Do $p_{i}={\left(p_{tr}\right)}_{i},i=1,N.$
      Else reject the step
    - Change β=1/T according to the selected scheduler.

As it is mentioned in [63] this search can get stuck in a local but not a global optimum. That is why the process is carried out several times, starting from different randomly generated matrices and saving the best result. Of course, we cannot guarantee that the optimum found is global. However, with a sufficiently slow descent and with a large number of simulations, one can hope to get good results. In case of large quantum systems, where the amount of parameters groves significantly, there are simply no other solutions. In this paper we used this algorithm, implemented on C++ language, to find the probability vectors that provide the maximum of *S* for the single qutrit ans the single ququad states. To this end the unitary matrix parametrization, described in the previous section, is used. The simulation was done as follows. 100 unitary rotation matrices were generated. For each matrix 10,000 MC steps were done, during which, the maximum value of *S* and the corresponding probability vector are found. The best probability vector that provides the “biggest maxima” among all the realisations was selected. Next, using the simulation results we guessed the exact maxima.

## 6. Results

### 6.1. Polygon Geometry of the Qutrit State

Let us introduce the single qutrit state, described by the 3×3 density matrix. The geometric portrait of this system is an equilateral eight angle polygon of equal sides $\sqrt{2}$ on the plane, formed by the set of simplexes (see Figure 6). Every side of the polygon is given by (14) and eight squares are constructed, with sides $y_{k}=A_{k}A_{k+1}$, associated with the polygon $A_{1}A_{2}\ldots A_{8}$ (see Figure 7). Using the MMC simulation one can estimate the maximum value of the sum (15) and the corresponding configuration of the probabilities. The computational results give that the maximal configuration is ${\hat{S}}_{max}=16+8\sqrt{2}=27.3126$, $p_{0}=p_{2}=p_{4}=p_{6}=0,\;p_{1}=p_{3}=p_{5}=p_{7}=1$, (see Figure 11). However, when the conditions on the eigenvalues of the density matrix (7) hold, the probabilities $p_{i}$ are connected by the positivity condition. The MMC algorithm must be changed. The unitary transformation, given by (18), is used to iterate through all the possible density matrices. The probabilities are recalculated at each MC step. We performed 100 simulations of 10000 Monte-Carlo steps. For each realization the maximum and the corresponding configuration of the spins are obtained. The configuration that provides the “biggest” maximum corresponds to the pure states. However, as we concluded for the single qubit system, different pure states provide different maxima of *S*. For example, using one of the results of the MMC realization, one can obtain $S_{q}=15.4748$, where $p_{0}=0.59503,$ $p_{1}=0.995853,$ $p_{2}=0.467081,$ $p_{3}=0.468792,$ $p_{4}=0.0871691,$ $p_{5}=0.0871691,$ $p_{6}=0.519888,$ $p_{7}=0.465404$. From the geometrical symmetry, we deduced the strict value ${\hat{S}}_{q}=10+9/\sqrt{2}=16.364$, where the probability configuration is $p_{0}=0,\;p_{1}=1,\;p_{i}=0.5,\;i\in 2,\ldots ,7$. The density matrix, corresponding to this configuration of probabilities, has three eigenvalues, λ={1,0,0}. Hence, these parameters provide the pure state. However, from 100 simulation, one can find a global maximum pure state, where $S_{qmax}=16.6228$ and $p_{0}=1,$ $p_{1}=\left(3-\sqrt{3}\right)/12,$ $p_{2}=\left(3+\sqrt{3}\right)/6,$ $p_{3}=\left(3+\sqrt{3}\right)/12,$ $p_{4}=p_{5}=p_{6}=p_{7}=0.5$. For the maximally mixed state $S_{mmix}=13.8005$, where $p_{0}=p_{1}=2/3,\;p_{i}=1/2,\;i\in 2,\ldots ,7$, hold. Similarly, the minima is $S_{min}=9+4\sqrt{2}=13.6569$, $p_{i}=0.5,\;i\in \left\{0,7\right\}$. Note, that the minima coincides both for the classical and for the quantum case.

Finally, the inequality for (15) is the following

(21) $$\begin{array}{ccc} S_{min}\leq & S & \leq S_{qmax}<S_{max},\\ 13.6569\leq & S & \leq 16.6228<27.3126. \end{array}$$

**Figure 8:**
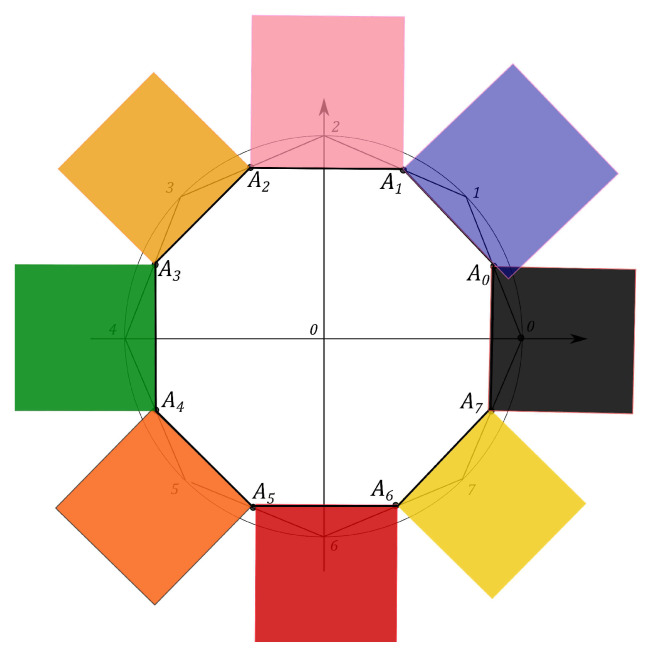
Malevich’s squares for the “minima” state, $S_{min}=13.6569$.

**Figure 9:**
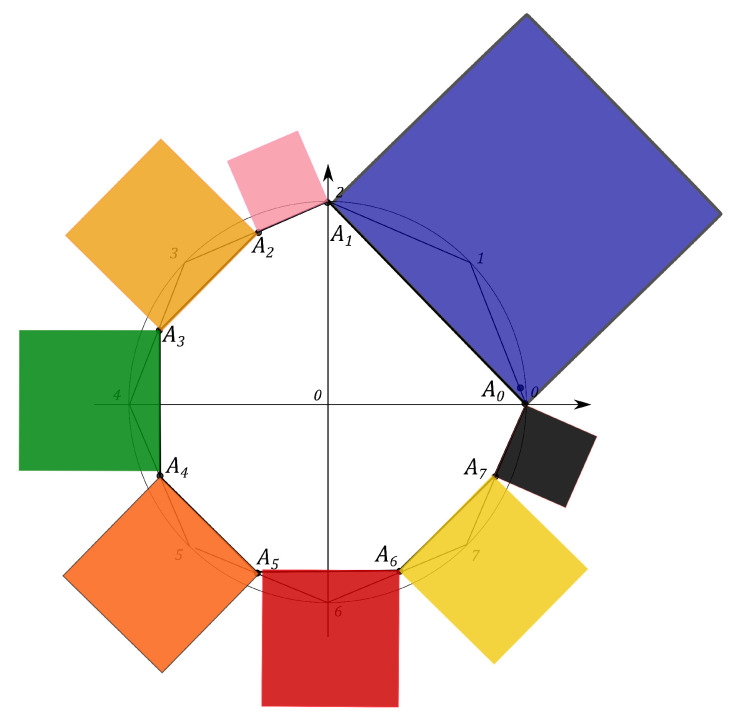
Malevich’s squares for the maximally mixed state, $S_{mmix}=13.8005$.

**Figure 10:**
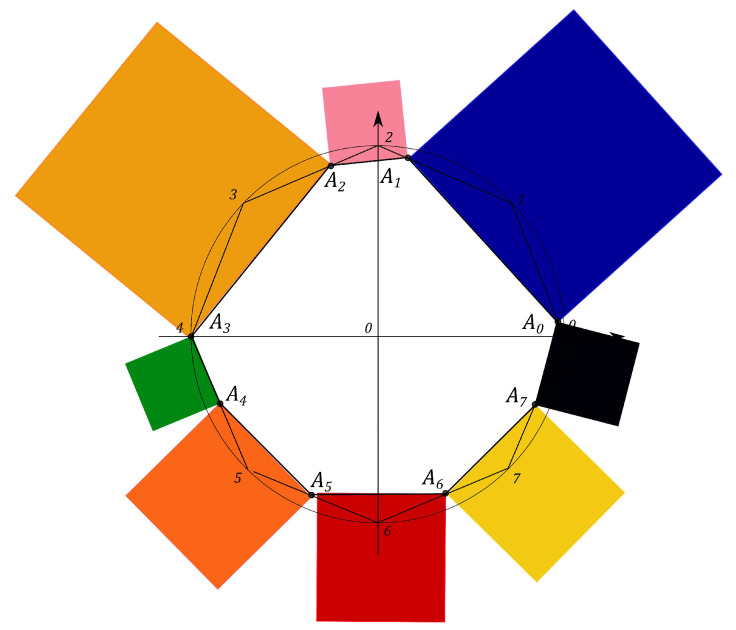
Malevich’s squares corresponding to “maximum” pure state, $S_{qmax}=16.6228$.

**Figure 11:**
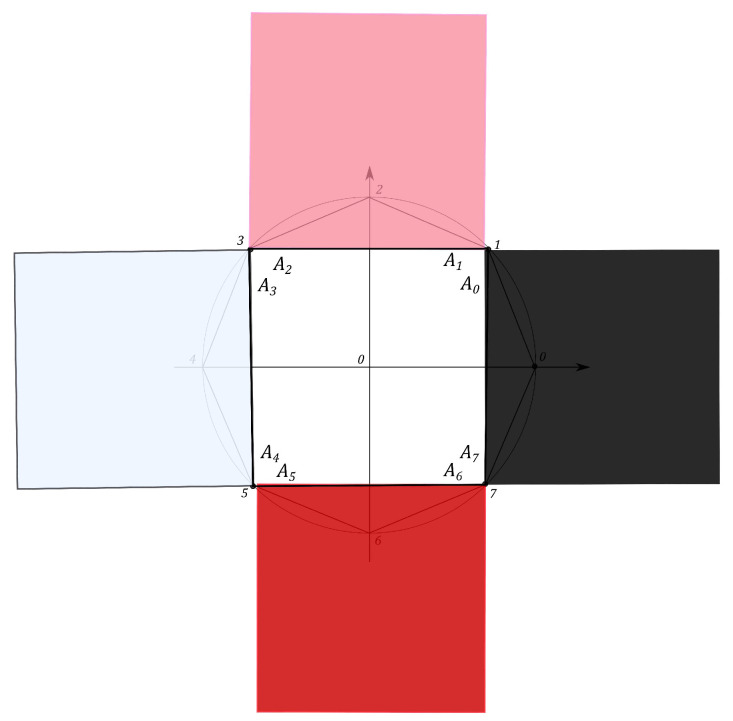
Malevich’s squares corresponding to classical case, ${\hat{S}}_{max}=27.3126$.

The Malevich’s squares, corresponding to the “minima” state, the maximally mixed state, the “maximum” pure state and the classical state, are shown in Figure 8, Figure 9, Figure 10 and Figure 11.

### 6.2. Polygon Geometry of the Ququad State

**Figure 12:**
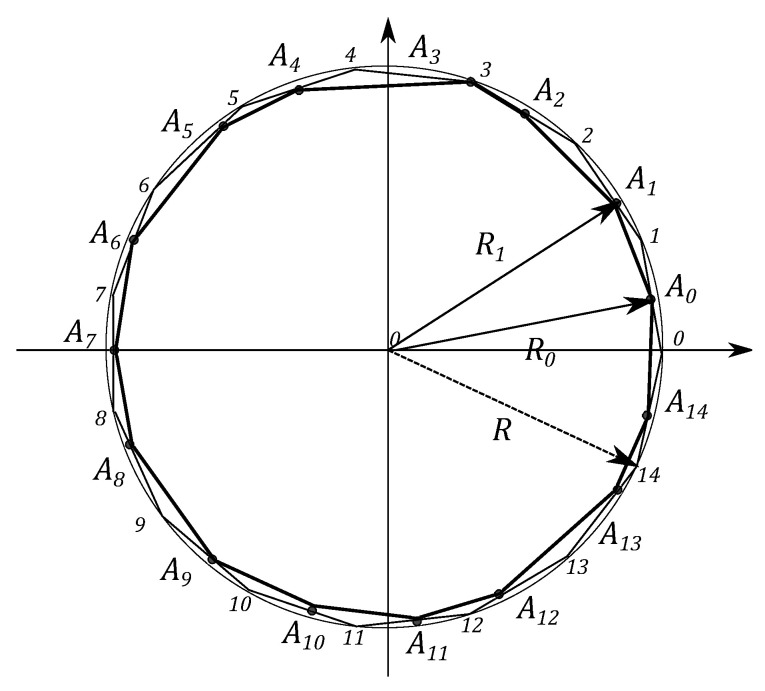
The 15 angle polygon with vertices $A_{k}$, k=0,…,14, determining the ququad state.

Similarly to the previous section to describe the single ququad state one can define N=15 simplexes and form from them the 15 angle equilateral polygon with the side lengths equal to $\sqrt{2}$. It can be divided by 15 sectors with an angle β=2π/15. One can connect points $A_{k}$, k=0,…,14, located on simplexes by lines and get the 15 angle polygon, presented in Figure 12.

**Figure 13:**
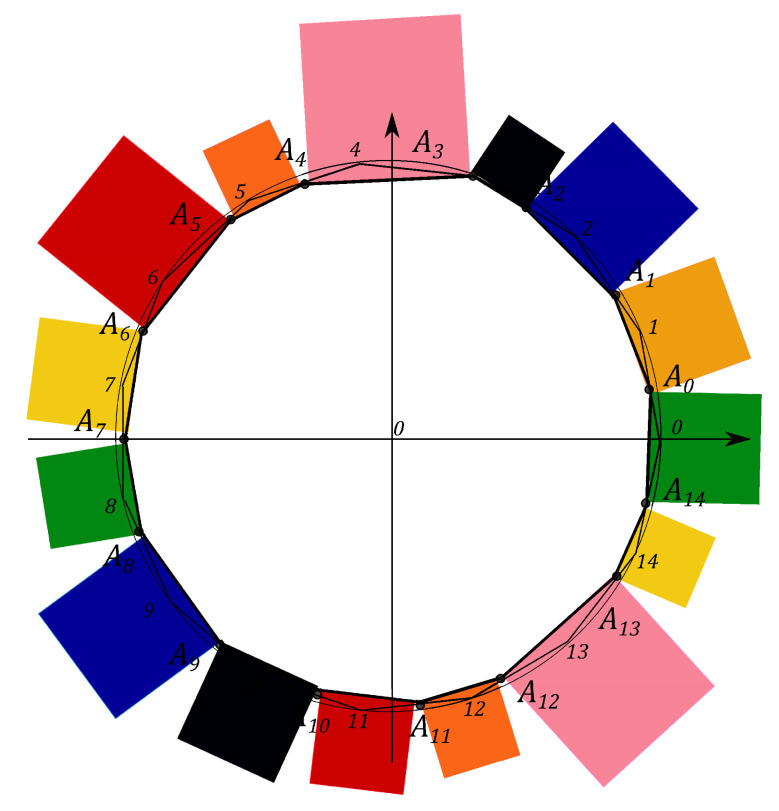
The polygon $A_{0}\ldots A_{14}$, determining the ququad state.

Fifteen squares are constructed, counterparts of the *Malevich’s Suprematist Composition*, with sides $y_{k}=A_{k}A_{k+1}$, associated with the polygon $A_{0}A_{1}\ldots A_{14}$ (see Figure 13). The sum of the areas is given by (15). Using the MMC simulation, one can find the maximum value of this sum and the corresponding configuration of probabilities. The computational results provide $S_{max}=30+28\cos\left(2\pi /15\right)=55.5793$ for $p_{2i}=0,\;p_{2i+1}=1,i\in \left[0,7\right]$. However, for the quantum case the conditions on the eigenvalues of the density matrix $\rho_{4}$, hold. Using the modified MMC algorithm, one can obtain ${\hat{S}}_{qmax}=30.8522$, $p_{0}=0.515446,$ $p_{1}=0.476783,$ $p_{2}=0.517941,$ $p_{3}=0.0554499,$ $p_{4}=0.532907,$ $p_{5}=0.299625,$ $p_{6}=0.531661,$ $p_{7}=0.519571,$ $p_{8}=0.373514,$ $p_{9}=0.69049,$ $p_{10}=0.667841,$ $p_{11}=0.547556,$ $p_{12}=0.369825,$ $p_{13}=0.847302,$ $p_{14}=0.476661$. From the geometrical symmetry, the strict maximum is $S_{qmax}=18+17\cos\left(2\pi /15\right)=33.5303$, where $p_{4}=1,p_{3}=p_{5}=0,p_{i}=1/2,i=\left\{0\ldots 14\setminus 3,4,5\right\}$. The density matrix, corresponding to the this configuration of probabilities, has four eigenvalues λ={1,0,0,0}. Hence, these parameters provide the pure state. The maximally mixed state provides $S_{mmix}=28.9964$, where $p_{3}=p_{4}=p_{5}=1/4,\;p_{i}=1/2,\;i\in \left\{0,\ldots ,14\setminus 3,4,5\right\}$. The minima is $S_{min}=28.7032$, $p_{j}=0.5$, j=0,…,14. Finally, the inequality is the following

$$\begin{array}{ccc} S_{min}\leq & S & \leq S_{qmax}<S_{max},\\ 28.7032\leq & S & \leq 33.5303<55.5793 \end{array}$$

### 6.3. Werner State in Probability Representation

One of the most important degraded Bell states is the Werner state [64]. As an example let us take the single qudit state with the spin j=3/2 and the Werner density matrix

$$\begin{array}{c} \rho_{W}=\frac{1-F}{3}I_{4}+\frac{4F-1}{3}|\psi^{-}\rangle \langle \psi^{-}|, \end{array}$$

where $I_{4}$ denotes the identity matrix, $|\psi^{-}\rangle$ is the singlet state of the Bell states. The Werner state is characterized by a single real parameter *F*, that measures the overlap of Werner state with the Bell state. If F≤1/2, the state is separable. The Werner state with $F>(2+3\sqrt{2})/8$ violates the Clauser-Horne-Shimony-Holt inequality. In terms of (5) the Werner density matrix elements can be written as

$$\begin{array}{c} p_{3}=\left(1-F\right)/3,p_{4,5}=\left(1+2F\right)/6,p_{12}=2\left(1-F\right)/3,\;p_{i}=1/2,\;i=\left\{0\ldots 14\setminus 3,4,5,12\right\}. \end{array}$$

Note that, unlike the general parametrization (5), some probabilities in the last formula are always equal. Thus, for the Werner state it will never reach the maximum or minimum of *S*. For F=0 the sum of the Malevich’s areas is S=29.2053 and the probability configuration is the following $p_{3}=1/3,\;p_{4}=1/6,\;p_{5}=1/6,\;p_{12}=2/3,$ $p_{i}=1/2,$ i={0…14∖3,4,5,12}. For F=1/2 the sum of the Malevich’s areas is S=29.1764 and $p_{3}=1/6,p_{4}=1/3,p_{5}=1/3,p_{12}=1/3,$ $p_{i}=1/2,$ i={0…14∖3,4,5,12}. For $F=(2+3\sqrt{2})/8$ the sum is S=29.8408 and $p_{3}=1/3\left(1-\left(2+3\sqrt{2}\right)/8\right),\;p_{4,5}=\left(2+\sqrt{2}\right)/8,p_{12}=\left(2-\sqrt{2}\right)/48,\;p_{i}=1/2,\;i=\left\{0\ldots 14\setminus 3,4,5,12\right\}$. The maximally mixed state is when F=1/4, holds. The sum is $S_{mmix}=28.9964$ and $p_{3}=1/4,\;p_{4}=1/4,\;p_{5}=1/4,$ $p_{i}=1/2,$ i={0…14∖3,4,5}. For F=1 the sum of the Malevich’s areas is $S_{qmax}=30.7032$ and $p_{3,12}=0,p_{i}=1/2,$ i={0…14∖3,12}. Finally, the inequality is

$$\begin{array}{ccc} S_{mmix}\leq & S & \leq S_{qmax},\\ 28.9964\leq & S & \leq 30.7032. \end{array}$$

**Figure 14:**
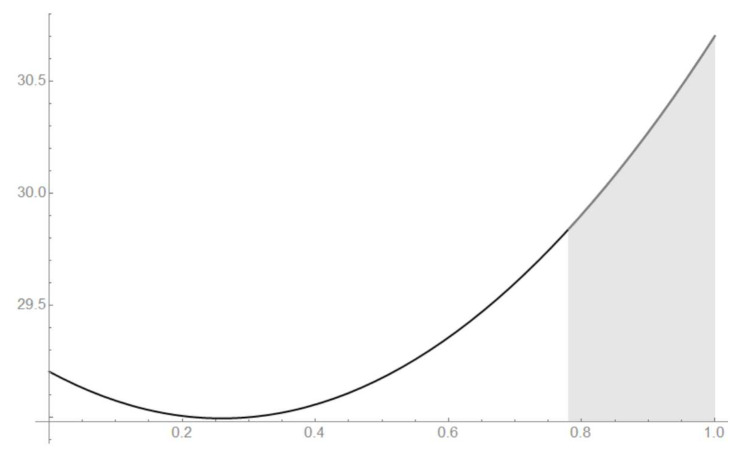
The sum of the Malevich’s square areas *S* for the Werner state.

These bounds can be seen in Figure 14. The maximally mixed state provides the minimum by *F*. The pure state, corresponding to F=1, provides the aper bound $S_{qmax}$.

## 7. Malevich’s Inequality Versus Uncertainty Relations

Uncertainty relation is one of the fundamental distinguishing features of quantum theory that lies in the heart of quantum mechanics and quantum information [4,65,66,67]. Kennard, Weyl, Robertson and Schrodinger derived several uncertainty relations, among which the most known one is the Heisenberg-Robertson relation

(22) $$\begin{array}{cc} & {\left(\Delta A\right)}^{2}{\left(\Delta B\right)}^{2}\geq |\frac{1}{2}\langle \Psi |\left[A,B\right]|\Psi \rangle {|}^{2}, \end{array}$$

where [A,B]=AB−BA, and the variances of observable *X* is defined by $\Delta X=\sqrt{\langle \Psi |X^{2}|\Psi \rangle -{\langle \Psi |X|\Psi \rangle }^{2}}$. The latter uncertainty relation states that the product of two variances of the measurement results of the incompatible observables is bounded by the expectation value of their commutator. A simple lower bound for the sum of the variances can be obtained from (22), using the fact that ${\left(\Delta A-\Delta B\right)}^{2}\geq 0$. Thus, one can write

(23) $$\begin{array}{cc} & {\left(\Delta A\right)}^{2}+{\left(\Delta B\right)}^{2}\geq 2\Delta A\Delta B\geq |\langle \Psi |\left[A,B\right]|\Psi \rangle |. \end{array}$$

Further we will use the notation 〈Ψ|[A,B]|Ψ〉≡〈[A,B]〉. In case of the three observables one can generalize (22) similarly to (23), namely

(24) $$\begin{array}{cc} & \Delta_{3}\geq \frac{1}{2}\left(\langle \left[A,B\right]\rangle +\langle \left[B,C\right]\rangle +\langle \left[C,A\right]\rangle \right), \end{array}$$

where $\Delta_{3}={\left(\Delta A\right)}^{2}+{\left(\Delta B\right)}^{2}+{\left(\Delta C\right)}^{2}$. In [51] two uncertainty relations for triple observables were proposed. They read as

(25) $$\begin{array}{cc} & \Delta_{3}\geq \frac{1}{3}\Delta {\left(A+B+C\right)}^{2}+\frac{1}{\sqrt{3}}\left|\langle \left[A,B,C\right]\rangle \right|, \end{array}$$

(26) $$\begin{array}{cc} & \Delta_{3}\geq \frac{1}{\sqrt{3}}\left(|\langle \left[A,B\right]\rangle \right|+\left|\langle \left[B,C\right]\rangle \right|+|\langle \left[A,C\right]\rangle |), \end{array}$$

where 〈[A,B,C]〉=〈[A,B]〉+〈[B,C]〉+〈[A,C]〉. The last relation is definitely stronger than (24). The Equation (25) is verified using single spin measurement in diamond (see [55]).

For the case of *N* incompatible observables $A_{1},A_{2},\ldots ,A_{N}$, the following variance-based uncertainty relation

(27) $$\begin{array}{ccc} \sum_{i=1}^{N}{\left(\Delta A_{i}\right)}^{2} & \geq & \frac{1}{2(N-1)}\sum_{1\leq i<j\leq N}{\left(\Delta (A_{i}+A_{j})\right)}^{2}, \end{array}$$

(28) $$\begin{array}{ccc} \sum_{i=1}^{N}{\left(\Delta A_{i}\right)}^{2} & \geq & \frac{1}{2(N-1)}\sum_{1\leq i<j\leq N}{\left(\Delta (A_{i}-A_{j})\right)}^{2}, \end{array}$$

hold. The second inequality was improved in [53], namely

(29) $$\begin{array}{ccc} \sum_{i=1}^{N}{\left(\Delta A_{i}\right)}^{2} & \geq & \frac{1}{N-2}\sum_{1\leq i<j\leq N}{\left(\Delta (A_{i}+A_{j})\right)}^{2}-\frac{1}{{\left(N-1\right)}^{2}\left(N-2\right)}{\left(\sum_{1\leq i<j\leq N}\Delta \left(A_{i}+A_{j}\right)\right)}^{2}. \end{array}$$

This inequality has a tighter lower bound then (27). In [52] a stronger uncertainty relation for *N* incompatible observables is deduced, i.e.,

(30) $$\begin{array}{ccc} \sum_{i=1}^{N}{\left(\Delta A_{i}\right)}^{2} & \geq & \frac{1}{N}{\left(\Delta \sum_{i=1}^{N}A_{i}\right)}^{2}+\frac{1}{N^{2}\left(N-1\right)}{\left(\sum_{1\leq i<j\leq N}\Delta \left(A_{i}-A_{j}\right)\right)}^{2} \end{array}$$

### 7.1. Measurements in Qubit System

Let us choose three Pauli operators as an example of incompatible observables

(31) $$\begin{array}{ccc} A & = & \sigma_{x},\;B=\sigma_{y},\;C=\sigma_{z}. \end{array}$$

The following variance

$$\begin{array}{c} \Delta \sigma_{i}^{2}=\langle \sigma_{i}^{2}\rangle -{\langle \sigma_{i}\rangle }^{2}=1-{\langle \sigma_{i}\rangle }^{2},\;i=x,y,z, \end{array}$$

holds. Using the notations provided in [54], namely

(32) $$\begin{array}{ccc} V & = & \;\;\;\;\;\;\sum_{i\in \{x,y,z\}}\;\;\;\;\;{\langle \sigma_{i}\rangle }^{2},D=\langle \sigma_{x}\rangle \langle \sigma_{y}\rangle +\langle \sigma_{y}\rangle \langle \sigma_{z}\rangle +\langle \sigma_{z}\rangle \langle \sigma_{x}\rangle ,\\ H & = & |\langle \sigma_{x}\rangle \left|+\right|\langle \sigma_{y}\rangle \left|+\right|\langle \sigma_{z}\rangle \left|,\;E=\right|\langle \sigma_{x}\rangle +\langle \sigma_{y}\rangle +\langle \sigma_{z}\rangle | \end{array}$$

one can rewrite the uncertainty relations (24)–(30) as

(33) $$\begin{array}{ccc} 3-V & \geq & H, \end{array}$$

(34) $$\begin{array}{ccc} 3-V & \geq & \sqrt{3}E-D, \end{array}$$

(35) $$\begin{array}{ccc} 3-V & \geq & \frac{2}{\sqrt{3}}H, \end{array}$$

(36) $$\begin{array}{ccc} 3-V & \geq & \frac{1}{2}\left(3-V-D\right), \end{array}$$

(37) $$\begin{array}{ccc} 3-V & \geq & \frac{1}{2}\left(3-V+D\right), \end{array}$$

(38) $$\begin{array}{ccc} 3-V & \geq & 2\left(3-V-D\right)-\frac{1}{4}{\left(L_{+}+M_{+}+N_{+}\right)}^{2}, \end{array}$$

(39) $$\begin{array}{ccc} 3-V & \geq & \frac{1}{3}\left(3-V-2D\right)+\frac{1}{9}{\left(L_{-}+M_{-}+N_{-}\right)}^{2}, \end{array}$$

where

(40) $$\begin{array}{ccc} L_{\pm } & = & \sqrt{2-{\left(\langle \sigma_{x}\rangle \pm \langle \sigma_{y}\rangle \right)}^{2}},\\ M_{\pm } & = & \sqrt{2-{\left(\langle \sigma_{y}\rangle \pm \langle \sigma_{z}\rangle \right)}^{2}},\\ N_{\pm } & = & \sqrt{2-{\left(\langle \sigma_{z}\rangle \pm \langle \sigma_{x}\rangle \right)}^{2}}. \end{array}$$

The latter relations were experimentally verified in [54] using the single-photon measurement experiments. The inequalities (35), (34) and (39) have more stringent bounds than others in qubit systems.

### 7.2. Relation of Classical Coin Probability Distribution and the Quantum Qubit States

It is known that the matrix elements of an arbitrary matrix can be related to some probability distribution. In [68] this fact is used to introduce an interesting quantization procedure of classical statistics. Following this line, we start from the classical coin tossing game. The aim of the game is that there is a classic coin, tossing which, the players get one of the two sides with a certain probability. For the loss of each side of the coin some reward is assigned. Using the classical probability distributions that describe the coin states, one can introduce the density matrices and the state vectors in the Hilbert space. The probability distributions are mapped onto the density operators, acting on the Hilbert space, and the classical random variables, used in a game as rewards, are mapped onto Hermitian matrices, namely the Hermitian operators, acting on the Hilbert space. Using this bijective mapping, one can rewrite the relations, known from quantum mechanics in the classical-like random variables and probability distributions.

As an example, let us start from the spin −1/2 state (qubit) described in Section 2. Since this matrix can be parametrized by three random variables, our classical coin tossing game consists from three non ideal coins. Their states are identified with three probability distributions $\left(p_{1},1-p_{1}\right)$, $\left(p_{2},1-p_{2}\right)$, $\left(p_{3},1-p_{3}\right)$, where $0\leq p_{1,2,3}\leq 1$ are the probabilities to have have the coin in “up” position. Hence, three random variables X(j), Y(j) and Z(j), j=1,2, can be introduced, such that X(1)=x, X(2)=−x, Y(1)=y, Y(2)=−y and $Z\left(1\right)=z_{1}$, $Z\left(2\right)=z_{2}$, where $x,y,z_{1},z_{2}$ are real numbers. The mean values and the moments of these random variables are the following

(41) $$\begin{array}{ccc} \langle X\rangle & = & xp_{1}-x\left(1-p_{1}\right),\;\langle Y\rangle =yp_{2}-y\left(1-p_{2}\right),\;\langle Z\rangle =z_{1}p_{3}+z_{2}\left(1-p_{3}\right). \end{array}$$

The numbers $p_{i}$, i=1,2,3 are organised the matrix form (1). Let the operator ${\hat{H}}_{2}$ have the Hermitian matrix, parametrized by three random variables, as

(42) $$\begin{array}{c} H_{2}=\left[\begin{array}{cc} z_{1} & x-iy\\ x+iy & z_{2} \end{array}\right]. \end{array}$$

The mean value is defined as

(43) $$\begin{array}{c} \langle H_{2}\rangle =Tr\left(\rho_{2}H_{2}\right)=\langle X\rangle +\langle Y\rangle +\langle Z\rangle . \end{array}$$

The Pauli matrices (31) correspond to (42) with the following parameters

$$\begin{array}{c} \sigma_{x},\;z_{1}=z_{2}=0,\;x=1,\;y=0;\\ \sigma_{y},\;z_{1}=z_{2}=0,\;x=0,\;y=1;\\ \sigma_{z},\;z_{1}=1,\;z_{2}=-1,\;x=0,\;y=0. \end{array}$$

One can conclude that the mean values of these observables are

(44) $$\begin{array}{c} \langle \sigma_{x}\rangle =2p_{1}-1,\langle \sigma_{y}\rangle =2p_{2}-1,\langle \sigma_{z}\rangle =2p_{3}-1. \end{array}$$

In these notations the uncertainty relation (1) can be written as follows

(45) $$\begin{array}{c} 3-V\geq 2. \end{array}$$

This uncertainty relation provides a quantitative description that only one spin component of a two-level system can have a well defined value. One can see, that this relation is the strongest among (33)–(39). As it is shown in [54] the inequalities (35), (34) and (39) most often reach the specified boundary.

Let us rewrite the sum of the areas of the three Malevich’s squares (11) in similar to (45) form, namely

$$\begin{array}{c} S=\frac{3}{2}+\;\;\sum_{i\in \{x,y,z}{\langle \sigma_{i}\rangle }^{2}+\frac{1}{2}\left(\langle \sigma_{x}\rangle \langle \sigma_{y}\rangle +\langle \sigma_{x}\rangle \langle \sigma_{z}\rangle +\langle \sigma_{z}\rangle \langle \sigma_{y}\rangle \right) \end{array}$$

or

(46) $$\begin{array}{c} S=\frac{3}{2}+V+\frac{D}{2}. \end{array}$$

Since (12), holds, one can rewrite (46) as

(47) $$\begin{array}{c} \frac{3}{2}+\frac{D}{2}\leq 3-V\leq 3+\frac{D}{2}. \end{array}$$

Using the Monte-Carlo algorithm, we considered $10^{4}$ different probability configurations $p_{i}$, i=1,2,3 and compared the lower and upper bounds of Malevich’s inequality (46) with the best inequalities (35), (34) and (39), that are verified experimentally in [54]. Malevich’s inequality reach the lower limit (45) for the probability configurations (13).

For the spin −1 system we select Weyl basis. The density matrix of spin −1 system can be parametrized by eight random variables and our classical coin tossing game consists from seven non ideal coins. Their states are identified with eight probability distributions $\left(p_{i},1-p_{i}\right)$, i=1,…,8, where $0\leq p_{i}\leq 1$ are the probabilities to have have the coin in “up” position. Hence, three random variables $X_{1,2,3}\left(j\right)$, $Y_{1,2,3}\left(j\right)$, j=1,2 and Z(i), j=1,2,3 can be introduced, such that $X_{1,2,3}\left(1\right)=x_{1,2,3}$, $X_{1,2,3}\left(2\right)=-x_{1,2,3}$, $Y_{1,2,3}\left(1\right)=y_{1,2,3}$, $Y_{1,2,3}\left(2\right)=-y_{1,2,3}$ and $Z\left(1\right)=z_{1}$, $Z\left(2\right)=z_{2}$, $Z\left(3\right)=z_{3}$ where $x_{1,2,3},y_{1,2,3},z_{1,2,3}$ are real numbers. The mean values and the moments of these random variables are the following

(48) $$\begin{array}{ccc} \langle X_{1}\rangle & = & x_{1}p_{3}-x_{1}\left(1-p_{3}\right),\;\langle Y_{1}\rangle =y_{1}p_{4}-y_{1}\left(1-p_{4}\right),\\ \langle X_{2}\rangle & = & x_{2}p_{7}-x_{2}\left(1-p_{7}\right),\;\langle Y_{2}\rangle =y_{2}p_{8}-y_{2}\left(1-p_{8}\right),\\ \langle X_{3}\rangle & = & x_{3}p_{5}-x_{3}\left(1-p_{5}\right),\;\langle Y_{3}\rangle =y_{3}p_{6}-y_{3}\left(1-p_{6}\right),\\ \langle Z\rangle & = & z_{1}\left(p_{1}+p_{2}-1\right)+z_{2}\left(1-p_{2}\right)+z_{3}\left(1-p_{1}\right). \end{array}$$

Let the operator ${\hat{H}}_{3}$ have the Hermitian matrix, parametrized by three random variables as

(49) $$\begin{array}{c} H_{3}=\left[\begin{array}{ccc} z_{1} & x_{1}-iy_{1} & x_{3}-iy_{3}\\ x_{1}+iy_{1} & z_{2} & x_{2}-iy_{2}\\ x_{3}+iy_{3} & x_{2}+iy_{2} & z_{3} \end{array}\right]. \end{array}$$

The mean value is defined as

$$\begin{array}{c} \langle H_{3}\rangle =Tr\left(\rho_{3}H_{3}\right)=\sum \langle X_{j}\rangle +\langle Y_{j}\rangle +\langle Z\rangle . \end{array}$$

Acting similar to the previous example, one can obtain

$$\begin{array}{c} \langle \sigma_{0}\rangle =2p_{3}-1,\;\langle \sigma_{3}\rangle =2p_{5}-1,\;\langle \sigma_{6}\rangle =2p_{7}-1,\;\langle \sigma_{1}\rangle =2p_{4}-1,\;\langle \sigma_{4}\rangle =2p_{6}-1,\\ \langle \sigma_{7}\rangle =2p_{8}-1,\;\langle \sigma_{2}\rangle =p_{1}+2p_{2}-2,\;\langle \sigma_{5}\rangle =p_{2}+2p_{1}-2. \end{array}$$

Hence, using this replacement, one can rewrite the Malevich’s inequality (22) for the single qutrit state in terms of the measurable quantities.

$$\begin{array}{ll} & 9+4\sqrt{2}\leq \frac{1}{3}(-2+3\left(\langle \sigma_{0}\rangle +\langle \sigma_{1}\rangle +\langle \sigma_{3}\rangle +\langle \sigma_{4}\rangle +\langle \sigma_{6}\rangle +\langle \sigma_{7}\rangle \right)\\ - & 2\left(\langle \sigma_{2}\rangle +\langle \sigma_{5}\rangle \right)+\frac{1}{2}\left(1+\frac{1}{\sqrt{2}}\right)({\left(1+\langle \sigma_{0}\rangle \right)}^{2}+{\left(1+\langle \sigma_{1}\rangle \right)}^{2}\\ + & {\left(1+\langle \sigma_{3}\rangle \right)}^{2}+{\left(1+\langle \sigma_{4}\rangle \right)}^{2}+{\left(1+\langle \sigma_{6}\rangle \right)}^{2}+{\left(1+\langle \sigma_{7}\rangle \right)}^{2})\\ + & \frac{4}{9}\left({\left(-2-2\langle \sigma_{2}\rangle +\langle \sigma_{5}\rangle \right)}^{2}+{\left(\langle \sigma_{2}\rangle -2\left(1+\langle \sigma_{5}\rangle \right)\right)}^{2}\right)\\ + & \frac{1}{3\sqrt{2}}(45+4\left({\langle \sigma_{2}\rangle }^{2}+{\langle \sigma_{5}\rangle }^{2}\right)+3({\langle \sigma_{3}\rangle }^{2}+{\langle \sigma_{4}\rangle }^{2}+{\langle \sigma_{6}\rangle }^{2}\\ + & {\langle \sigma_{7}\rangle }^{2})+12\left(\langle \sigma_{3}\rangle +\langle \sigma_{4}\rangle \right)+3(\langle \sigma_{1}\rangle \langle \sigma_{3}\rangle +\langle \sigma_{3}\rangle \langle \sigma_{4}\rangle \\ + & \langle \sigma_{4}\rangle \langle \sigma_{6}\rangle +\langle \sigma_{6}\rangle \langle \sigma_{7}\rangle -\langle \sigma_{0}\rangle \langle \sigma_{1}\rangle )+\langle \sigma_{0}\rangle (1+4\langle \sigma_{2}\rangle \\ - & 2\langle \sigma_{5}\rangle )+10\langle \sigma_{5}\rangle +12\langle \sigma_{6}\rangle +13\langle \sigma_{7}\rangle +4\langle \sigma_{5}\rangle \langle \sigma_{7}\rangle \\ - & 2\langle \sigma_{2}\rangle \left(-5+2\langle \sigma_{5}\rangle +\langle \sigma_{7}\rangle \right))\leq \frac{1}{4}\left(40+17\sqrt{2}+\sqrt{6}\right). \end{array}$$

The resulting inequality can be experimentally verified and compared, for example with (30).

## 8. Discussion and Conclusions

In this paper we introduced the universal geometrical picture of the single qudit quantum system, based on the new quantum suprematism approach, introduced in [44,45,46]. The single qubit is identified with the set of three probability distributions of the spin projections on the three perpendicular directions in space. Three probabilities, parametrizing the density matrix of the qubit, form the three simplexes on the plane. Connecting them, the triangle is formed, the sides of which serve as bases for the three squares, analogues of the Malevich’s squares. The sum of the areas of these squares characterize the quantum properties of the single qudit. In this paper, we proposed to go further and introduce polygons, built on simplexes, formed by the probabilities, that parametrize the density matrix, to describe the higher dimension quantum systems. The sides of the polygon serve as bases for the set of squares that form an analogue of the Malevich’s Suprematist Composition. The universal inequality, based on the sum of the areas of the squares, is constructed. The upper bounds of the inequality is different for the classical set of probabilities and for the quantum system with the density matrix, parametrized by the probability set. The inequality illustrates the single qudit states quantumness. In quantum case, the upper bound of the inequality corresponds to the pure states as the surface of the Bloch sphere for the single qubit states. The upper bound of the inequality can be found for the qudit states of high dimensions, for example, using the Metropolis Monte-Carlo algorithm. In contrast to the known multidimensional geometrical representations, the Malevich’s Suprematist Composition provides a simple and beautiful picture on the plane. The results are illustrated by the example of the single qutrit and ququad states. Particular attention is paid to the case of the Werner ququad state. Finally it is shown that the Malevich’s inequalities can serve as uncertainty relations for the systems of high dimensions and successfully compete with already known uncertainty relations for many arbitrary incompatible observables. It is expected that the multi-observable Malevich’s inequalities can be verified experimentally using the techniques similar to ones that are presented in [54,55].
